# Adaptive Potential of *Syzygium maire*, a Critically Threatened Habitat Specialist Tree Species in Aotearoa New Zealand

**DOI:** 10.1111/eva.70161

**Published:** 2025-10-02

**Authors:** Colan G. Balkwill, Emily Koot, Peter Ritchie, David Chagné, Julie R. Deslippe

**Affiliations:** ^1^ School of Biological Sciences Victoria University of Wellington Wellington New Zealand (Aotearoa); ^2^ The New Zealand Institute for Plant and Food Research Limited Auckland New Zealand (Aotearoa); ^3^ Genomics Aotearoa Palmerston North New Zealand (Aotearoa)

## Abstract

The restoration of swampland is vital for the recovery of both biodiversity and cultural values in Aotearoa New Zealand. *Syzygium maire*, an endemic wetland tree species, is a focus of many wetland restoration efforts. Formerly widespread, extant populations are small, fragmented, and under pressure from myrtle rust. Restoration initiatives may be unknowingly compounding these threats to the species by failing to represent the complete genetic diversity of populations. What genetic diversity remains in remnants and how it is distributed is not known. We therefore aimed to assess the national scale population structure, genetic diversity, and adaptive potential of *S. maire* to inform species conservation. We identified over 760,000 high‐quality single nucleotide variants in 269 reproductive age trees from across the species' range, using low coverage whole genome resequencing. At a national scale, we found five distinct regional‐scale genetic clusters, which in turn exhibit local structure and admixture. In the North Island: Northland, Bay of Plenty in the central east, Taranaki in the central west, and Greater Wellington/Manawatū in the south. A single cluster was identified in the South Island, Marlborough. Within‐cluster substructure was particularly evident for Greater Wellington/Manawatū. Genetic diversity and fixation indices (*F*_ST_) were relatively uniform across all clusters, and there was some evidence of north to south increase in kinship and shorter time since radiation. These patterns are likely to reflect glaciation cycles that resulted in complex contractions into local microrefugia and subsequent re‐radiations of the species over time. Genotype by environment analysis detected genetic variants potentially contributing to environmental adaptation, notably precipitation seasonality. Restoration and conservation goals would best be served by capturing diversity within regional clusters. Information on the geographic and environmentally structured distribution of this tree's genetic diversity supports conservation and restoration strategies through ensuring the complete extant diversity is captured, identifying regions at most risk of genetic degradation, and facilitating planning regarding the movement of adaptive diversity in a changing environment.

## 1 Introduction

*Syzygium* is the most speciose tree genus, comprising over 1000 recognized species (Govaerts et al. 2008; Low et al. 2022). *Syzygium maire* ( *A. Gunn* .) Sykes et Garn.‐Jones (swamp maire, maire tawake, waiwaka) is endemic and the only *Syzygium* species native to Aotearoa New Zealand (Dawson et al. 2011; Mahuta et al. 2021). The genus *Syzygium* is an important food source for birds, mammals, and insects and a structural component of many ecosystems, especially across the tropical Indo‐Pacific (Parnell et al. 2007).

Occurrence patterns and habitat suitability modelling suggest *S. maire* inhabited swampland across much of the North Island and the northern tip of the South Island before modern widescale deforestation (McCarthy et al. 2019). Its primary habitats are wetlands with a low water table, stream gullies, and, at higher elevations, cloud forest (Clarkson 2014; McCarthy et al. 2019; Mahuta et al. 2021; de Lange 2024). Pneumatophores (aerial roots) enable it to grow in waterlogged soils. In cases where water tables are continuously high, *S. maire* forms a dominant component of swamp forests, often together with pukatea (*Laurelia novae‐zelandiae*), a more widespread, generalist species. *S. maire* provides structural habitat and shelter for some of the country's most threatened fish species (Mahuta et al. 2021). The tree's fruits are also a food source for larger birds such as kererū ( *Hemiphaga novaeseelandiae* ), tūī ( *Prosthemadera novaeseelandiae* ), and korimako ( *Anthornis melanura* ) (Clarkson 2014; van der Walt et al. 2020). Generation time and maximum age for the species are unknown, but young saplings in the order of five to ten years old have been observed fruiting.

Wetlands are taonga (treasured) for Māori who use them for food (kai), medicine (rongoā), and material resources (Pa Ropata McGowan 2021). Prior to European settlement in the late 19th century and widespread changes to land use, wetland coverage in Aotearoa was approximately 2.4 million ha or 9% of the total land area (Dymond et al. 2021). Much of this would have been habitat for *S. maire*. Large tracts of the lowland North Island (Te Ika‐a‐Māui) had extensive and potentially interconnected wetlands. For instance, early European farmers in the central east North Island (Taranaki) described farms where 74 ha of a 76 ha lot consisted of swamp land (Clarkson 2014). Ninety percent of this total area has since been lost to anthropogenic activity, particularly drainage for pasture, with over 95% lost in the North Island (Dymond et al. 2021). Most wetlands are now small and surrounded by non‐native land cover, likely leading to significant fragmentation and further degradation (Myers et al. 2013). As a result of habitat loss and disruption to dispersal, a large proportion of wetland plant and vertebrate species are threatened (de Lange et al. 2009; Clarkson et al. 2013; Dunn et al. 2018), and ecosystem services are disrupted (Tomscha et al. 2021; Bentley et al. 2022; Tomscha et al. 2023).

Threats to *S. maire* result from habitat loss, fragmentation, and novel disease pressure. When plant population size decreases, there is likely to be an increase in inbreeding and genetic drift, leading to reduced adaptive genetic diversity and accumulated deleterious mutations (Slotte et al. 2010; Kutschera et al. 2020). Concurrently, high levels of fragmentation can lead to functionally isolated subpopulations instead of larger, interbreeding metapopulations, further reducing effective population size (Pflüger et al. 2019). Large tracts of swampland have been drained, and where trees remain, ground saturation is often too inconsistent for seedling establishment (van der Walt et al. 2020). Seeds do not persist within the seedbank from season to season. Rather, they germinate within 6–8 weeks or lose viability (van der Walt et al. 2020). Once genetic diversity is lost from an area, therefore, it cannot be recovered. Self‐fertilization and reliance on dispersal by animals, which are presently absent or threatened, are likely compounding the negative genetic effects of fragmentation on *S. maire* (Anderson et al. 2011; Van Etten et al. 2015; Rodger et al. 2021). Furthermore, fragmentation effects are amplified in patches surrounded by markedly different land use because dispersers are far less likely to move across transformed landscapes, an observation that is particularly pertinent for wetlands (Schlaepfer et al. 2018). Many *S. maire* seeds are not dispersed far from their mother tree (van der Walt et al. 2020), and bird dispersers often remain close to their place of feeding (Wotton and Kelly 2012). Furthermore, insect pollinators can differentially affect gene flow depending on species but are not likely to move great distances while foraging (Brunet et al. 2019). Strong familial relationships among trees close together in a forest are therefore likely, further increasing the chance of inbreeding in small and isolated patches. Finally, the species is severely affected by myrtle rust (*Austropuccinia psidii*) (de Lange et al. 2024), a fungus originating from South America that attacks leaves, flowers, and fruit of a diverse range of species in the family Myrtaceae (Coutinho et al. 1998; Glen et al. 2007). Infection can leave trees unable to produce new growth and render them functionally sterile (Pegg et al. 2014). Regeneration will therefore require managed conservation and restoration efforts relating to both habitat and population‐level genetics (Herbert et al. 2025).

Restoration planting in Aotearoa is an active part of conservation (Norton et al. 2018). Germination and establishment of *S. maire* in nurseries has been well‐developed and vegetative propagation is not a significant challenge (Bettoni et al. 2023). However, information on how to structure planting programmes and where to obtain seed is lacking. The spatial structure of the remaining genetic diversity across a species' range is an important consideration in successful conservation and restoration planning (Andrello et al. 2023; Wei et al. 2023). Based on biogeographic boundaries and population genetics analyses of several native tree species, Heenan et al. (2023) described nine broad seed sourcing zones across Aotearoa which span entire regions of tens of thousands of square kilometres. Native species studied to date have low to medium genetic diversity and differentiation (Heenan et al. 2022; Koot et al. 2022; Chagné et al. 2023; Heenan et al. 2024; McCartney et al. 2024). All these tree species, however, have different dispersal mechanisms and life histories from *S. maire*, as they are primarily insect‐pollinated with wind‐dispersed seed. Moreover, they are all habitat generalists and unlikely to have experienced the extreme habitat loss and fragmentation that *S. maire* has. At the same time, strict seed sourcing zones do not necessarily account for environmental change (Heenan et al. 2023). Species‐specific information on population structure, diversity, and adaptive variation is required to design sound conservation strategies.

In this study, we aim to characterize national‐scale population genetic structure and genetic diversity of *S. maire* to inform conservation and restoration decisions for the species. As fragmentation is relatively recent and trees are long‐lived, we expect genetic signatures to be representative of natural rather than anthropogenic processes. We expect that most of the diversity will exist in the northern part of the species' range because it is likely to have arrived first and persisted through glacial cycles there. Finally, we evaluate adaptive variation in the species, which may facilitate range shift or mitigate maladaptation to a changing climate. We discuss these findings in the context of the unique biogeographical history of Aotearoa and make recommendations for species conservation.

## 2 Methods

### 2.1 Sample Collection

**FIGURE 1:**
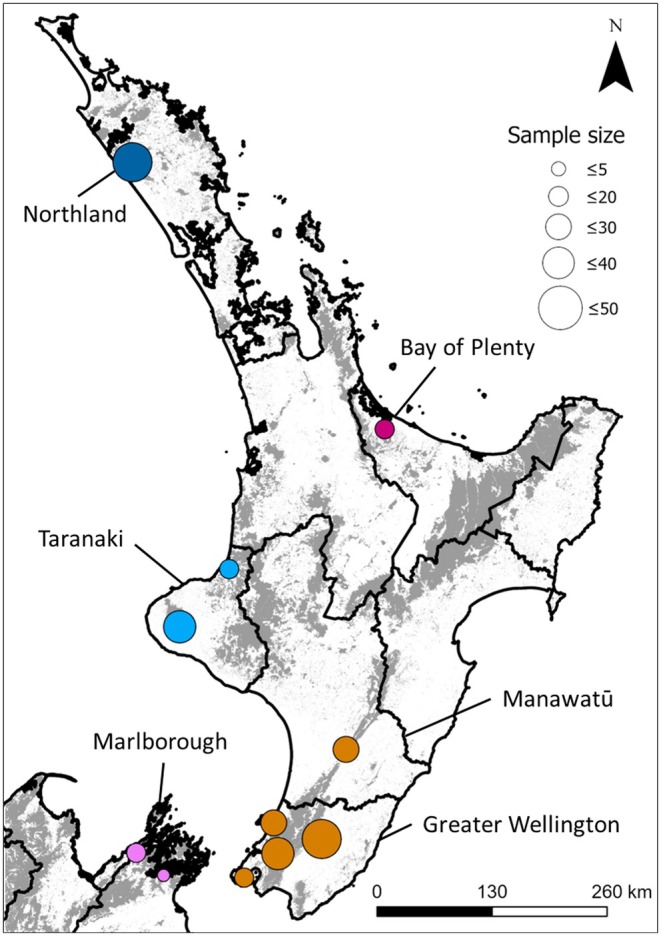
Locations of sampling sites used for this study. Each circle represents multiple smaller sampling sites (see Table S3 for a breakdown of sampling sites per region). Circle size reflects sample size, while its color corresponds to the assigned genetic cluster according to *k*‐means analysis. Dark lines represent political borders, while light gray relief represents the remaining native forest. Landcover information was derived from the New Zealand Landcover Database version 5 (LRIS), geographical borders from NZ Coastlines and Islands Polygons (LINZ), and political boundaries from the generalized regional councils 2023 layer (StatsNZ).

Whole leaf samples were collected from adult trees between November 2020 and April 2022. When provided, culturally informed sampling protocols were followed. Where possible, young leaf tissue was harvested, but due to accessibility issues (tall trees, high water levels, or thick bush), it was often necessary to harvest mature leaves. As far as possible, samples were obtained from across the species' range (Figure 1 and Table S1), but some were omitted from sampling due to COVID‐19 or myrtle rust restrictions. Overall, 32 sampling sites across six regions were visited. Ten to twenty individuals were sampled at each study site. Where more than 20 adult trees were present, individuals were selected from across the site to minimize overrepresentation of highly related individuals. As *S. maire* individuals often have multiple trunks and grow close together, leaves were only collected from a single trunk if multiple boles occurred within a square meter. GPS coordinates and diameter‐at‐breast height were recorded for all samples.

### 2.2 Low‐Coverage Whole Genome Resequencing and Variant Calling

DNA was extracted from leaf tissue using a modified CTAB protocol (Balkwill et al. 2024). Dried DNA samples (> 500 ng where possible) were sent to Neogen, Lincoln, NE, USA (https://www.neogen.com/) in two separate batches, initially in May 2021 and then in August 2022. 150 bp paired‐end libraries were prepared using the Illumina Nextera Kit. Sequencing was performed on an Illumina NovaSeq instrument at an average of 10× coverage per sample. Neogen conducted initial quality filtering, returning only samples with a score of Q30 at over 75% of bases.

Sequencing read quality was assessed using FastQC v0.11.9 (Andrews 2010) and multiQC v1.9 (Ewels et al. 2016). We then trimmed reads using fastp v0.23.2 (Chen et al. 2018) with adapter detection, overrepresentation analysis, base correction for mismatched bases in paired reads, and removal of low‐quality sequences enabled. Next, we mapped the samples to all scaffolds in the *S. maire* reference genome (Balkwill et al. 2024) using bwa‐mem2 (Li 2013) in SAMtools v1.15.1 (Li et al. 2009). The resulting BAM files were filtered for a quality score of 20 (99% accuracy) using SAMtools *view*, mate pairs cleaned with SAMtools *fixmate*, and sorted with SAMtools *sort*. To remove PCR duplicates from the mapped reads, we used Picard MarkDuplicates (version 2.7.1; https://broadinstitute.github.io/picard/), followed by indexing with SAMtools *index*. We performed indel realignment using GATK v3.5 (McKenna et al. 2010). Target intervals of interest were generated from sample alignments using RealignerTargetCreator and used to inform IndelRealigner in generating a BAM file with marked duplicates. Alignment statistics were calculated using SAMtools *stats, flagstat*, *coverage*, and *depth* and visualized in R v4.2.1 (R Core Team 2013). Variants for individual scaffolds were detected using BCFtools v1.15.1 (*mpileup* followed by *call* with multiallelic calling enabled) against the unmasked *S. maire* reference genome and called with BCFtools v1.19 (Danecek et al. 2021). Finally, variant files for each scaffold were concatenated into a single file using *bcftools concat*.

In order to set cut‐offs for filtering high‐quality variants and minimize errors while maximizing retained data, we assessed mean depth and missing information per individual and per site, variant quality score, and allele frequency using VCFtools v0.1.15 (Danecek et al. 2011). Individuals with a mean depth of < 3% or > 20% missing information were removed from the dataset and variant calling was re‐performed as above re‐performed with only passing samples. Per‐site cutoffs were set to a mean depth of five, a maximum depth of 12 (twice the mean coverage), a minimum quality of 50, and maximum missing information of 10%. Furthermore, only biallelic and non‐indel sites were included. Multiple minor allele frequency (MAF) thresholds were used depending on the analysis (Table S2). Filtering was performed using VCFtools.

### 2.3 Linkage Disequilibrium, Outlier Allele, and Relatedness Filters

We chose to filter for linkage disequilibrium (LD) as it is possible that high levels may result in groupings that reflect genotypic association rather than true genetic structure (Price et al. 2008; Abdellaoui et al. 2013). To filter for LD, we calculated the linkage decay for each chromosome in PLINK v1.09 (Purcell et al. 2007). We excluded variants with a genotyping rate below 90% and individuals with a genotyping rate below 50%. Linkage disequilibrium was then calculated as the squared correlation coefficient (*r* ^*2*^) on a sliding window of 100 variants with a physical distance of 1000 kb. Variants were not filtered based on *r* ^*2*^, and all variants were included. We used information from the linkage decay to identify an *r* ^2^ cutoff according to the background value of *r* ^2^ for the genome. A cutoff was explored at two stringency levels, 0.1 as the most stringent and 0.2 as less stringent. A list of single nucleotide variants (SNPs) exceeding these thresholds was generated using PLINK with sliding windows of 50 kb sliding by 10 kb. VCFtools was then used to remove linked SNPs.

Outlier SNPs were detected in order to identify putative neutral and non‐neutrally segregating genetic variation through pcadapt v4.3.3 (Luu et al. 2017). The method assumes SNPs that contribute exceptionally to population structure are under positive selection, ascertained through principal component analysis (PCA) and Mahalanobis distance. We chose this method because it is fast (given our large dataset), and we had no prior knowledge of the level of admixture, true population structure, and the demographic history of the species. All are variables that can affect *F*_ST_ (model) based measures, but to which pcadapt is robust (Luu et al. 2017). The methods followed were those suggested on the package website (https://bcm‐uga.github.io/pcadapt/articles/pcadapt.html). In order to determine an appropriate number of explanatory principal components, we assessed the scree plot for each dataset and used Cattell's rule to determine the cutoff for K (Cattell 1966). We also examined the score plots to visually ascertain at which PCA component population structure was no longer obvious. Significance cut‐offs for outlier detection were applied through *q*‐value with alpha set to 0.1 using the R package *q*value v2.30.0 (Dabney and Storey 2011).

For the admixture and genotype environment association, related individuals were removed from the dataset using KING version 2.3.2 (Manichaikul et al. 2010). For all pairs of individuals with relatedness greater than or equal to 0.125 (second cousins), one was removed from the analysis.

### 2.4 Genetic Structure

#### 2.4.1 Non‐Parametric Approaches

To identify genetic clusters within the data, we used *k*‐means clustering, PCA, and discriminant analysis of principal components (DAPC). We chose to assess the effect of varying MAF to account for the potential loss of information on population structure with more stringent MAF and data filtering (Linck and Battey 2019). PCA was run on datasets filtered for three values of MAF: 0.00, 0.025, and 0.05. We began by replacing missing SNP information according to mean allele frequencies before running PCA with the *dudi.pca* function from the R package ade4 v1.7‐22 (Dray and Dufour 2007). We set the number of kept axes to the number of individuals (269) but did not normalize column vectors. In addition to the three levels of MAF filtering, we also ran the PCA on the same datasets filtered for LD. The remaining analyses were performed on MAF 0.05 filtered for linkage disequilibrium (Table S2). *K*‐means clustering was performed with *find.cluster* in adegenet v2.1.10 (Jombart 2008) in R. We set the maximum number of clusters to 20 and the number of principal components to the number of individuals in the analysis (269). The choice of the optimal number of clusters was made interactively based on the Bayesian Information Criterion (BIC) score. As a final clustering method, we ran DAPC (Jombart et al. 2010). DAPC is an extension of PCA in that it maximizes the variance between groups or clusters while minimizing within‐group variance. The method thereby focuses on intergroup differences rather than differences between all individuals. Prior groups for DAPC were those identified by *k*‐means clustering. For DAPC, the number of principal components was again set to the number of individuals (269). All discriminant functions were retained as the number was small (≤ 5). To reduce the number of principal components and overfitting while maintaining a sufficient number for discrimination between groups, we used the *optim.a.score* function and reran DAPC with the number of principal components set to that which generated the best *a*‐score.

#### 2.4.2 Parametric, Model‐Based Approaches

We assessed admixture using ADMIXTURE (Alexander and Lange 2011). We used the most stringent MAF for this analysis (0.05), filtered for LD, outlier alleles, and related individuals to ensure inferences were not affected by undersampled variation and that model assumptions were not violated.

ADMIXTURE v1.3.0 was run on default settings. We performed 5‐fold cross‐validation and assessed admixture between 2 and 12 ancestral populations. The best value of K was that which minimized the cross‐validation error.

To explore potential lineage mixing alongside shared ancestry of populations, we constructed neighbour joining networks implemented in SplitsTree v6.1.16 (Huson 1998), which allows for the representation of conflicting dendrograms simultaneously (Bryant and Moulton 2004). Calculations were based on the MAF0.05 dataset filtered for LD, with and without outliers. For the distance matrix, we calculated pairwise Nei's D (Nei 1972) with the R package StAMPP v1.6.3 (Pembleton et al. 2013). Distance matrices were then imported into SplitsTree, where the NeighbourNet algorithm was used to construct unrooted neighbour‐joining phylogenetic networks. Neighbour‐joining trees were also reconstructed with the SplitsTree software and visualized in Figtree v1.4.4.

### 2.5 Summary Statistics

All estimates were performed using the R package *hierfstat* v0.5.11 (Goudet 2005). Observed, expected, and total heterozygosity, as well as fixation index (*F*_ST_) and inbreeding coefficient (*F*_IS_), were calculated for the MAF0.05 dataset including and excluding outliers, using the methods of Nei (1987) and *basic_stats* function. *F*_ST_ and *F*_IS_ were also estimated for these datasets using Weir and Cockerham (1984)'s statistics with *wc*. Population‐specific *F*_ST_ and *F*_IS_, as well as within and between population gene diversity, were estimated with bootstrap confidence intervals using *betas* and 1000 bootstraps (Weir and Goudet 2017; Goudet et al. 2018). Population was set to sampling region. Pairwise *F*_ST_ and confidence intervals for all population pairs were calculated using Weir and Cockerham (1984)'s methods with *genet.dist* and *boot.ppfst* with 1000 bootstraps.

### 2.6 Kinship and Inbreeding

Inter‐individual kinship coefficients were calculated according to Weir and Goudet (2017) using the hierfstat function *beta.dosage*. These measures account for many individuals in our study having relatively high kinship and levels of inbreeding, small populations, and that individual trees were spaced at distances smaller than the potential dispersal distance, violating the major assumption of independence of samples, which is necessary for reliable estimates from many other methodologies (Weir and Goudet 2017, Goudet et al. 2018). Therefore, these methods are particularly useful for studies on rare and endangered species. The methods of Goudet et al. (2018) and Weir and Goudet (2017) also increase in accuracy with a large number (at least > 10,000) SNPs. By filtering for rare alleles, the accuracy of kinship estimators is reduced (Goudet et al. 2018). Calculations were therefore performed on the linkage disequilibrium filtered MAF0.00 dataset.

### 2.7 Genotype Environment Association Analysis

Genotype environment association (GEA) analyses were run on the MAF0.05 datasets filtered for linkage disequilibrium and related individuals. Environmental layers were obtained from the New Zealand Environmental Data Stack (NZEnvDS) v1.1 (Leathwick 2002; Roudier et al. 2020; McCarthy et al. 2021). We excluded variables with Pearson's correlations above 0.7, variables which were not thought to be of importance to adaptability (e.g., distance to road) and soil pH due to missing data at some sites. We calculated allele frequencies using the vcfR v1.15.0 function *AD_frequency* (Knaus and Grünwald 2017). Partial redundancy analysis (pRDA) was performed in vegan v2.6‐6.1 (Oksanen et al. 2016) in R. Initially, the model was run on all retained environmental variables. In order to further reduce collinearity, the variable with the highest variance inflation factor was sequentially removed and the model rerun until all values were three or less (Zuur et al. 2010). The remaining environmental variables were then included as a conditioning factor in the model. To account for population structure, we used the first three PCs of the equivalent outlier filtered dataset as a conditioning variable in a partial RDA. We chose to retain RDA axes by assessing scree plots of their eigenvalues and the statistical significance of each axis according to the *anova.cca* function in vegan. SNPs associated with each RDA axis were identified and filtered according to standard deviation from the mean loading for each axis. We chose standard deviations of 2.5 and 3 as cutoffs. The correlation of each outlier SNP to each environmental variable was used to assign SNP's adaptive effect. Latent factor mixed models (LFMM) were run in the R package lfmm v1.1 (Caye et al. 2019) using the *lfmm_ridge* function. We set *K* = 5 as the number of genetic clusters identified by *k*‐means analysis. The algorithm was run for a maximum of 100 iterations for each environmental factor. To correct for multiple testing, genomic inflation factor adjusted *p*‐values were converted to *q*‐values using the package *q*‐value (Dabney and Storey 2011) and significant SNPs filtered at a false discovery rate (FDR) of 0.1 and 0.05. Overlapping SNPs identified by RDA and LFMM for each environmental variable were then compared to outliers detected by pcadapt. SNPs were retained if identified as outliers by all three methods, to ensure the lowest possible number of false positives (Forester et al. 2018).

### 2.8 Data Sovereignty

The *S. maire* specimens used in this study were from the traditional lands of numerous mana whenua iwi (tribes) across Te Ika‐a‐Māui/North Island and Te Waipounamu (South Island) of Aotearoa New Zealand (Table S1). Therefore, these people are the rightful guardians of the *S. maire* specimens used in this study, including raw and analyzed data derived from them. Authority over their *S. maire* gives effect to the Treaty of Waitangi, a treaty signed in 1840 between Māori chiefs and the British Crown. Internationally, Māori ownership and control of natural resources are supported by the UN Declaration on the Rights of Indigenous Peoples 2007. The Treaty of Waitangi and genomics research ethics in Aotearoa are discussed further by Morgan et al. (2019), Hudson and Russell (2009), and Collier‐Robinson et al. (2019). Biocultural (BC) Labels and Notices are being submitted by iwi and the authors, respectively, through the Local Contexts Hub (localcontexts.org) and the Aotearoa Genomics Data Repository under project ID “e2e9341e‐bdd3‐4078‐b230‐4f8acd25c6b0”. Where this is not already known, iwi are being contacted to determine what level of management they would like to exercise over the data generated in this study.

## 3 Results

### 3.1 Sequencing and Variant Calling

In total, 298 samples were sequenced. Of these, 29 were removed from the analyses due to low call rate resulting from low sequencing quality. We successfully sequenced and called variants for 269 *S. maire* trees distributed across the species' range (Figure 1 and Table S1). Prior to filtering, we identified 3,770,953 variants (Table S2). Most minor alleles occurred at a frequency below 0.03, with peaks around 0.05 and 0.02 and a long tail beginning around 0.15 (Figure S1). After applying quality filters, we retained 1,914,938 and 764,344 SNPs for the MAF0.00 and 0.05 datasets, respectively (Table S2). The percentage of missing data was 0.86% and 0.77% for these datasets, respectively. Mean depth per SNP varied between 6.26 for the unfiltered dataset and 7.19 for the dataset filtered for a MAF of 0.05 but not linkage disequilibrium. Individual level depth and missing data varied between 3.00 and 21.28 depth and 0.01%–5.97% missing data.

SNPs were not uniformly distributed across the *S. maire* genome, with high‐density regions (up to 36.15 per kb) within and surrounding some putative centromeres and chromosome ends (Figure S2). This was not the case for every putative centromeric region, however, nor did high density always seem to be associated with repeat regions. Outside of these regions of high SNP density, variants were more evenly distributed.

Linkage disequilibrium varied across chromosomes, with the highest *r* ^*2*^ values observed for chromosome IW04 (Figure S3). In general, linkage decay was a function of genetic distance, with most linkage decaying to the genomic background (approximately 0.01) within 250 kb. Some chromosomes, however, exhibited long‐range linkage disequilibrium (> 750 kb in this case). This was not detected at more stringent MAF filtering levels.

For outlier filtering, PCA eigenvalue cutoffs for explanatory versus random variation were not obvious, with any number between three and five approximating Cattell's rule in this analysis (Figure S4). Cattell's rule specifies that eigenvalues contributing to non‐random variation fall to the left of the straight line when the eigenvalue is plotted against percentage variation explained in a scree plot (Cattell 1966). We chose *K* = 5 PCA axes to retain for all datasets.

For the admixture and GEA analyses, we detected 137 sample pairs with kinship ≥ 0.125 (second cousins) and removed 66 individual trees accordingly. The final dataset for these analyses comprised 203 individuals (Table S1).

### 3.2 Genetic Structure and Admixture

PCA identified clustering within sampling regions for all levels of MAF and linkage disequilibrium filtering (Figure S5). Explanatory power for axis one varied between 6.5% and 9.0%, and axis two varied between 4.5% and 6.6%. For the dataset filtered for a MAF of 0.05 and linkage disequilibrium at *r* ^*2*^ of 0.2, explanatory power for axis one and axis two was 5.3% and 7.9%, respectively. Four to five distinct groups were evident for all datasets: Northland (NOR), Taranaki/Bay of Plenty (TAR/BOP), Manawatū/Greater Wellington (MAN/GWE), and Marlborough (MAR). Within clusters, substructure was evident, with samples collected in subregions clustering closer together. The first axis appeared to primarily explain differences in north–south variation among groups, with MAR at one extreme, followed by southern North Island groups (GWE/MAN), central North Island groups (TAR/BOP), and finally northernmost (NOR) groups at the other. Axis two captured diversity relating to the separation of MAR and GWE/MAN populations from the others, but did not resolve variation relating to the separation of NOR, TAR, and BOP. This axis also seemed to explain some within‐population diversity.

Filtering at different MAF and linkage disequilibrium levels did not substantially change the clustering, apart from the separation between northern (NOR) and central North Island (TAR/BOP) groups becoming less distinct at higher levels of MAF (Figure S5). We therefore chose the more stringent MAF 0.05 dataset filtered for linkage disequilibrium at an *r* ^*2*^ of 0.2 for downstream analysis, unless otherwise specified (Table S2). Reconstruction of PCA on the outlier filtered set resulted in the collapse of the dataset into two clusters—north (NOR/BOP/TAR) and south (MAR/GWE/MAN) (Figure S6).

**FIGURE 2:**
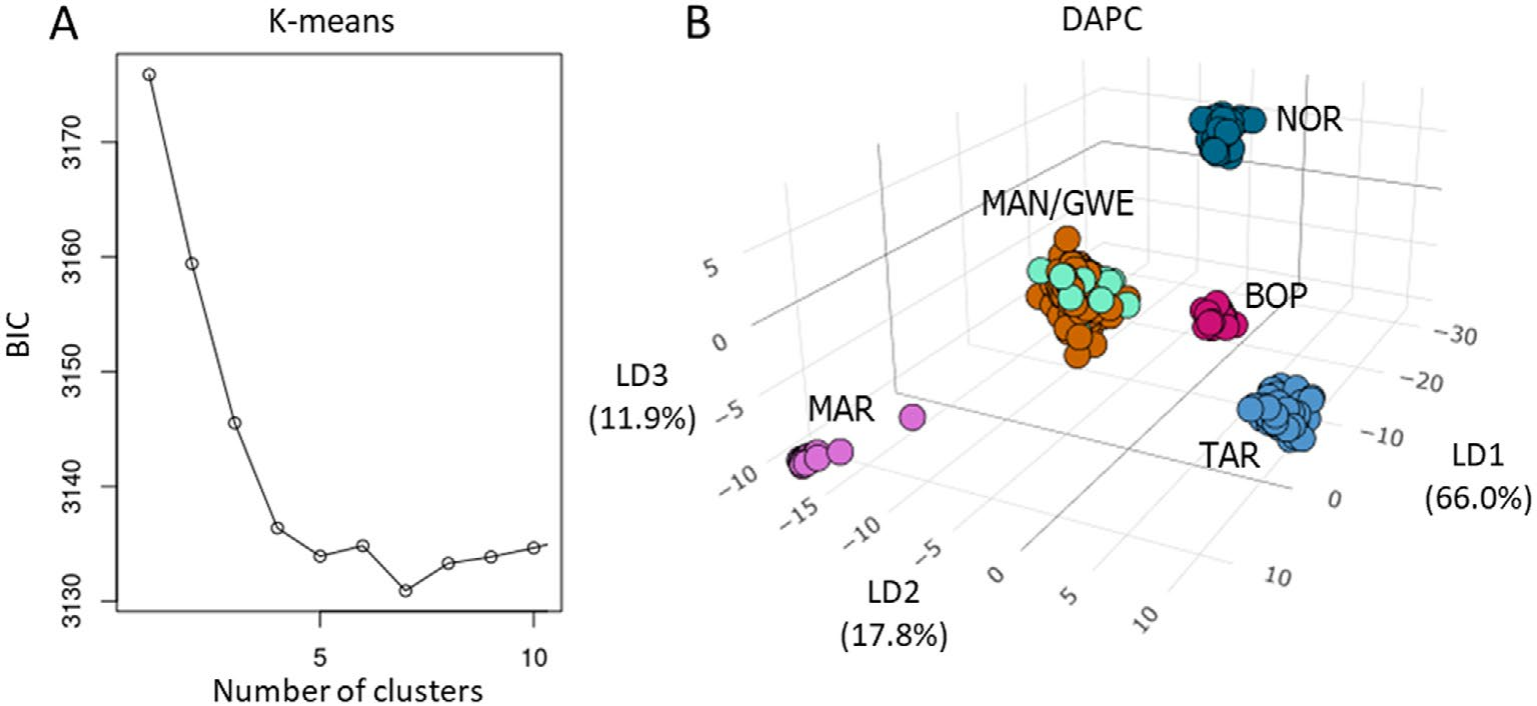
Clustering analysis of 269 *S. maire* trees sampled across Aotearoa. Individuals are coloured according to the region from which they were sampled, as follows: Northland (NOR), Bay of Plenty (BOP), Taranaki (TAR), Manawatū (MAN), Greater Wellington (GWE) and Marlborough (MAR). (A) *K*‐means clustering analysis. Calculations were performed for 20 values of K, but only the first 10 are shown here. Optimal values for K are the first minimum Bayesian Information Criterion (BIC) that occurs before a subsequent increase in BIC. Hence, optimal number of clusters for this analysis was 5. (B) Three‐dimensional representation of discriminant analysis of principal components (DAPC) for a dataset of 188,131 SNPs. Each point depicts a single individual. LD1 explains 66.0% of the variation in the dataset, and largely separates northern and southern populations. LD2 explains 17.8% of the variation, and appears to segregate MAR and TAR from the other populations.

*K*‐means clustering performed on 20 successive analyses suggested the existence of five main clusters (Figures 1 and 2A), which broadly corresponded to the same clusters identified by PCA. *K*‐means differed in splitting the TAR/BOP cluster into two according to region. MAN and GWE were again predicted to belong to the same genetic group.

DAPC identified the same major clusters as the *k*‐means analyses (Figure 2B), and 66.0% of variation was explained by LD1. This axis again explained the difference between the southern (MAR/GWE/MAN), central (TAR/BOP), and northern (NOR) populations. Variation between TAR and the remaining populations was largely explained by LD2 (17.8%), and LD3 (11.9%) explained the variation between MAR and TAR and the remaining populations, as well as some of the variation in the BOP. Despite this evidence that GWE and MAN are not separate populations genetically, they are isolated from each other and of separate conservation interest due to cultural values. Our discussion and recommendations therefore treat them separately.

**FIGURE 3:**
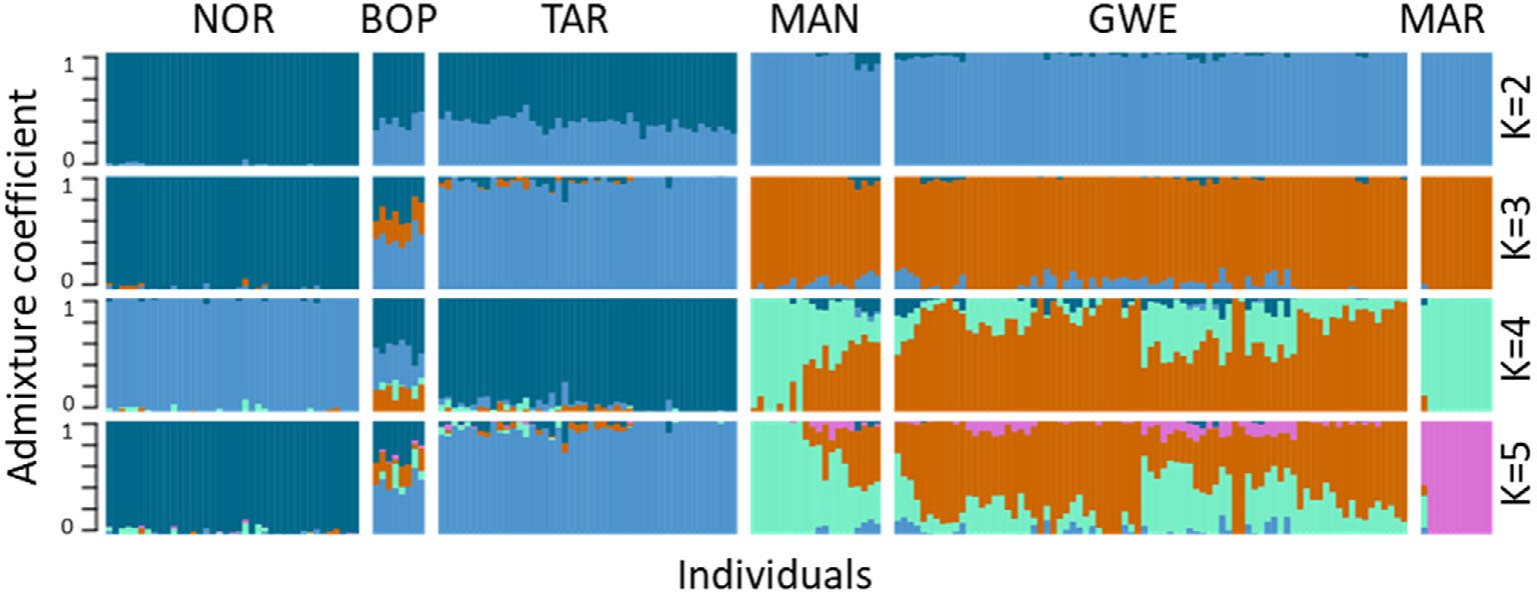
Admixture analysis for 203 individual trees sampled across Aotearoa. To generate the plot, ADMIXTURE was run for a dataset of 188,131 SNPs filtered for a minor allele frequency of 0.05, linkage disequilibrium, and related individuals. The outcome is an estimation of the proportion of ancestry for each individual derived from a number of hypothetical ancestral populations (as defined by *K*). Individuals are organized according to region from north at the left of the plot to southern populations at the right. We assessed the shared ancestor for two to seven common ancestors; the size and colour of each bar reflect the proportional contribution of an ancestral population to that individual. Abbreviations for regions are: Northland (NOR), Bay of Plenty (BOP), Taranaki (TAR), Manawatū (MAN), Greater Wellington (GWE), and Marlborough (MAR).

For the ADMIXTURE analysis, the value of K that minimized the cross‐validation error was two for both the outlier filtered and unfiltered datasets. For the unfiltered dataset at *K* = 2, there was a north–south divide, with NOR forming one cluster and MAN/GWE/MAR forming another, and BOP/TAR showing a mixture of the two (Figure 3). At *K* = 3, TAR clearly segregates, with BOP showing an intermediate position between all three. At *K* = 4, increased structure in GWE and MAR becomes evident. MAR becomes distinct at *K* = 5. These trends are largely similar up until *K* = 7, at which point BOP populations split from the central North Island cluster (data not shown). The outlier filtered dataset yielded similar trends (Figure S7), especially in terms of overall structure, but exhibited higher levels of admixture across all values of *K*, except for MAR. The neighbour‐net analyses grouped samples into six main clusters (Figure S8). Structure based on previously identified clusters was evident, with proximity roughly corresponding to genetic relatedness. The major GWE/MAN cluster was split into two, with MAR placed in between. They also appear to be derived from only a few founders and are closely related, as is represented by the tight clustering of nodes along branches and minimal occupation of graph space. In general, the GWE/MAN cluster did not appear to have a strict tree shape. The overall structure is clade‐structured, with defined medium to long branches between the southern clusters and central and northernmost clusters. The topology around all branch points for all other defined clusters is clade‐structured, with obvious genetic separation between regions. Within regions, however, most samples seem to radiate from the same tightly defined clusters of splits. Most uncertainty among regions and sub‐regions exists early on in lineages. Distance in terminal branches also far exceeds that between regions.

The neighbour‐joining tree (Figure S9) reiterates many of these observations. In most cases, terminal branches are long, with internal branches near the split of most major regions occurring much further back in evolutionary time. In the case of NOR, TAR, and GWE/MAN in particular, terminal branches are often far longer than any internal branches. In the case of BOP and MAR trees, internal branches are shorter, with longer internal branches. The GWE/MAN cluster has not been resolved perfectly, with individuals from one site grouped with individuals from another site. Of note, the node from which the MAR samples branch from the GWE/MAN clades seems to be as distant as the common ancestors for many of the GWE branches.

### 3.3 Summary Statistics

Overall *F*_ST_ across multiple methods was between 0.14 and 0.15 (0.03 for the outlier filtered set) when calculated for the clusters identified by *k*‐means clustering. Observed, expected, and total heterozygosity were 0.17, 0.19, and 0.22 (0.17, 0.16, 0.18 with outliers removed), respectively (Table 1).

**TABLE 1:** Global population genetic statistics for all loci across all individuals.

	*H*_o_	*H*_s_	*H*_t_	*F*_ST_	*F*_IS_
Nei (1987)	0.17 (0.17)	0.19 (0.16)	0.22 (0.18)	0.15 (0.03)	0.08 (−0.03)
Weir and Cockerham (1984)	—	—	—	0.14 (0.03)	0.10 (−0.03)

*Note:* Values in parentheses indicate those calculated for the outlier filtered dataset. *F*_IS_ = inbreeding coefficient; *F*_ST_ = fixation index; *H*_O_ = observed heterozygosity; *H*_S_ = expected heterozygosity within subpopulations; *H*_t_ = total expected heterozygosity.

**FIGURE 4:**
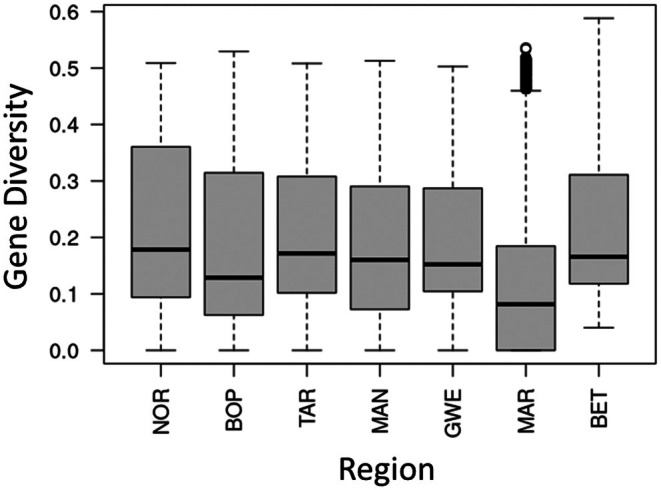
Gene diversity (heterozygosity) for each sampling region. Boxplots represent the median and interquartile range of values, with whiskers depicting the 95% confidence intervals of heterozygosity and outliers depicted as black dots. Mean values of heterozygosity are depicted by +. Statistics were calculated on 188,131 SNPs filtered for linkage disequilibrium and minor allele frequency of 0.05. BET, between population diversity; BOP, Bay of Plenty; GWE, Greater Wellington; MAN, Manawatū; MAR, Marlborough; NOR, Northland; TAR, Taranaki.

Population‐specific inbreeding and *F*_ST_ were lowest in NOR, followed by TAR, GWE, MAN, BOP, and by far the highest in MAR (Table 2). The overall *F*_ST_ was 0.17. Inbreeding values were more uniform across the populations than *F*_ST_ but still varied from 0.06 for NOR to 0.12 for MAN and MAR (Table 2). Removing outliers generally reduced differences in *F*_ST_ among populations for all but MAR, which still had a far higher value than others. Gene diversity varied across populations, where the highest mean diversity was within the NOR (0.22) and TAR (0.21) clusters, followed by GWE and MAN (0.20) and BOP (0.19), and the lowest MAR (0.12) (Figure 4). The mean between‐population diversity was 0.23. With outliers removed, the mean variation within populations fell between 0.18 (NOR) and 0.12 (MAN and MAR), with a between‐population *F*_ST_ of 0.17 (Figure S10).

**TABLE 2:** Population‐specific and overall *F*_IS_ and *F*_ST_ with confidence intervals.

	*F*_ST_	*F*_ST2.5%CI_	*F*_ST97.5%CI_	*F*_IS_
Northland	0.02 (−0.05)	0.02 (−0.05)	0.03 (−0.04)	0.06 (−0.04)
Bay of Plenty	0.18 (0.05)	0.17 (0.05)	0.18 (−0.06)	0.11 (−0.04)
Taranaki	0.07 (−0.05)	0.07 (−0.06)	0.07 (−0.05)	0.09 (−0.02)
Manawatū	0.14 (0.03)	0.14 (0.03)	0.14 (−0.03)	0.12 (−0.03)
Greater Wellington	0.10 (−0.02)	0.10 (−0.02)	0.10 (−0.02)	0.10 (−0.04)
Marlborough	0.49 (0.25)	0.48 (0.25)	0.49 (0.26)	0.12 (−0.03)
All	0.17 (0.04)	0.17 (0.04)	0.17 (0.04)	0.10 (−0.03)

*Note:* Values in parentheses indicate those calculated for the outlier filtered dataset. *F*_IS_ = inbreeding coefficient; *F*_ST_ = fixation index.

Pairwise *F*_ST_ between populations did not reflect any obvious north–south trend (Table S3). Rather, all populations were most different from the MAR samples, ranging from 0.32 for BOP—MAR to 0.19 for GWE—MAR. The most similar regions were again GWE and MAN with an *F*_ST_ of 0.05. All other values fell between 0.10 and 0.15, with the highest values delineating northern and southern clusters. Although *F*_ST_ values were far lower (≤ 0.07) with outliers removed, the same trends were observed.

### 3.4 Kinship and Inbreeding

**FIGURE 5:**
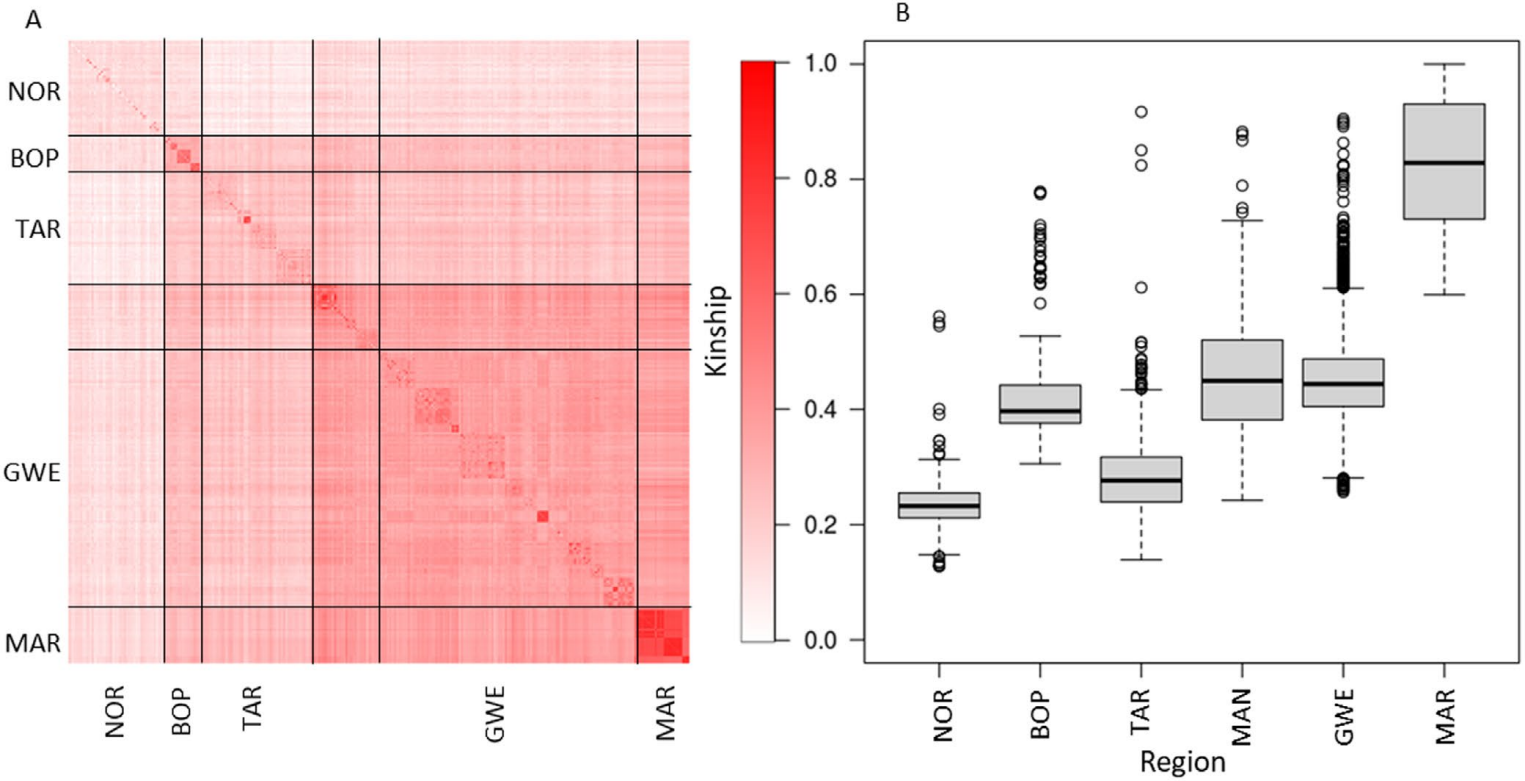
Kinship of all individuals and regions. (A) Heatmap representing scaled pairwise individual kinship. Calculations were performed in hierfstat and based on 957,330 SNPs filtered for linkage disequilibrium but not minor allele frequency. Inbreeding coefficients are displayed on the diagonal. All values were scaled to the minimum in the dataset. Individuals are grouped into regions from which they were sampled, and subregions are arranged from north to south. (B) Boxplots showing the distribution (median, interquartile range, 95% confidence intervals, with outliers depicted as single unfilled dots) of scaled beta‐kinship values per region. BOP, Bay of Plenty; GWE, Greater Wellington; MAN, Manawatū; MAR, Marlborough; NOR, Northland; TAR, Taranaki.

**FIGURE 6:**
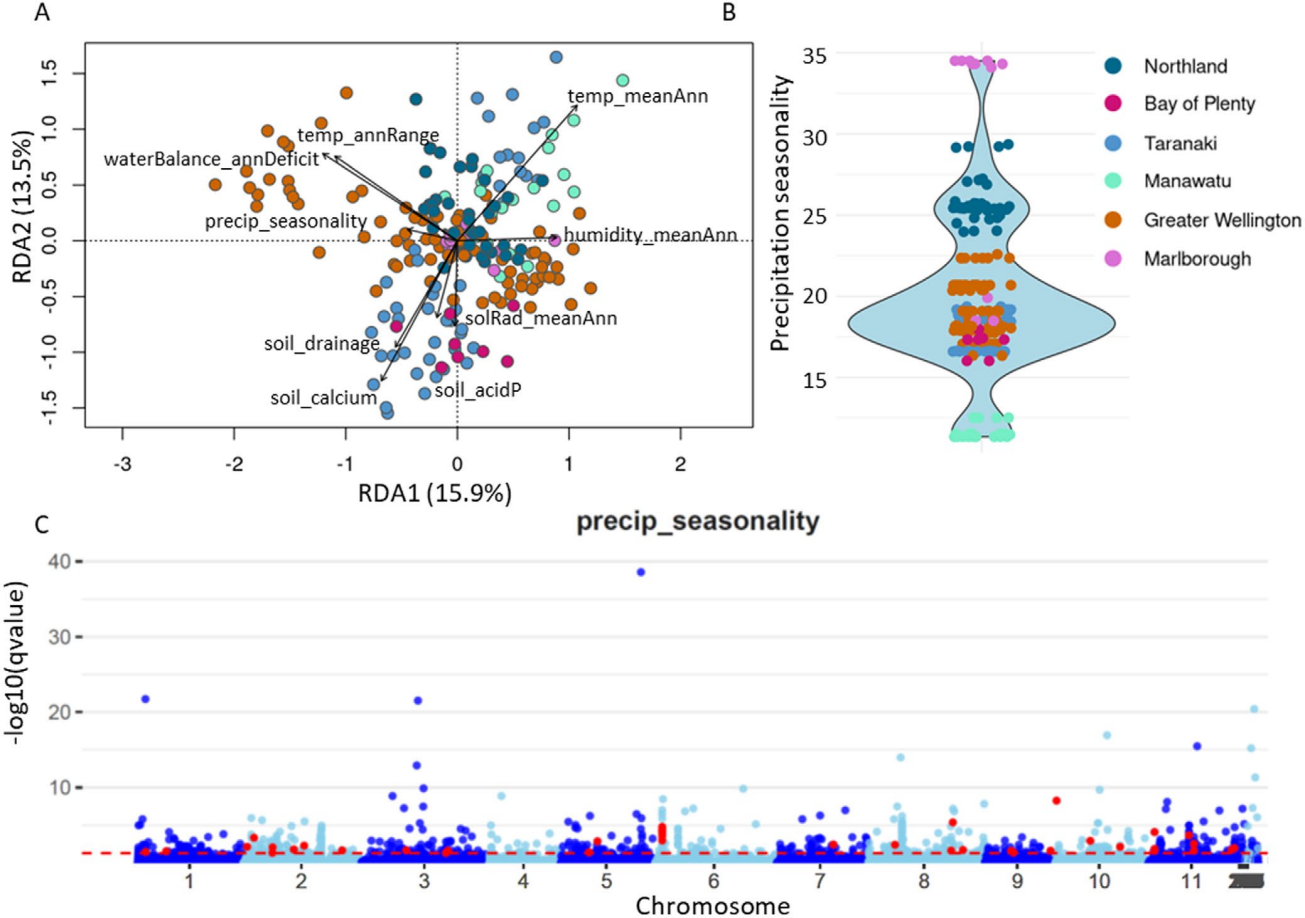
Genotype by environment analysis for precipitation seasonality. (A) RDA axes one and three for SNPs; circles denote individual trees, and environmental variables are overlain as vectors. The length and direction of vectors is proportional to the strength of the relationship of each environmental predictor and the SNPS. Individuals are coloured according to sampling region: Northland (NOR), Bay of Plenty (BOP), Taranaki (TAR), Manawatū (MAN), Greater Wellington (GWE) and Marlborough (MAR). (B) Violin plot depicting data quantity along the gradient of assessed precipitation seasonality. Precipitation seasonality is measured as the ratio of the standard deviation of the monthly total precipitation to the mean monthly precipitation. Each point represents the seasonality at an individual sample location. (C) Manhattan plot of LFMM analysis. The significance of each SNP's association (−log10 of the *q*‐value) with precipitation seasonality is shown on the *y*‐axis. Chromosome number and the physical position of SNPs are shown on the *x*‐axis. A *q*‐value cutoff of 0.05 (red dashed line) was chosen. SNPs depicted in red were also identified by the RDA analysis and present in the outlier set detected by pcadapt.

For the kinship analysis, relatedness appeared to increase southward in blocks corresponding to the broad clusters identified previously (Figure 5). Trees in NOR had the lowest regional kinship and the lowest kinship with other regions (Figure 5B). This was followed by TAR and BOP populations, then by the GWE/MAN cluster. MAR trees exhibited the highest within‐cluster kinship and were all similarly related to GWE/MAN individuals. Smaller blocks with high inter‐individual kinship are evident within all populations and generally correspond to sampling sites (Figure 6A). This local structure seems to be far lower in the TAR and NOR populations. Local relatedness is present in BOP, but kinship between blocks is low. This low between‐sampling site kinship breaks down to some extent in the GWE/MAN cluster, where higher kinship between sites is more evident for some individuals. The MAR population shows high kinship apart from a few individuals. These are also the more admixed, genetically diverse individuals that appear to be more closely related to North Island populations.

Inbreeding was lowest in NOR, followed by TAR, similar for BOP, GWE, and MAN, and highest in MAR (Figures 5A and S11). Apart from MAR, a wide range of inbreeding values was observed. All except one individual in MAR had inbreeding values near or outside of the upper bounds for all other regions.

### 3.5 GEA Analysis

Ten environmental variables were retained after filtering for inter‐variable correlation and variance inflation: mean annual solar radiation, mean annual temperature, precipitation seasonality, annual temperature range, annual water deficit, mean annual humidity, topographical position, soil calcium, soil drainage, and soil phosphorus (Figure S12). We chose to use the first three axes for the RDA analysis (Figures 6 and S13). ANOVA reported all axes as significant at *p* < 0.001, but the first three contained the majority of variance. Adjusted *r* ^*2*^ indicated that the full model explained 13.2% of the variation, with climate and population structure contributing 5.5% and 4.7%, respectively (Table S4). RDA analysis with thresholds at 2.5 and 3 SD discovered 12,371 and 4942 SNPs, respectively (Table S5). Of these, most were associated with soil calcium, followed by annual deficit in water balance. LFMM identified 3432 and 1954 environmentally correlated SNPs at cutoffs for 0.1 and 0.05 FDR. The majority were associated with precipitation seasonality, then mean solar radiation. When common SNPs were identified between the two analyses, 688 and 246 were retained. Most were associated with mean annual water deficit and precipitation seasonality (Table S5, Figures 6, S14, and S15). Finally, upon removing PCA outliers, 417 and 151 final candidate SNPs were identified depending on the stringency of filters. For the most stringent filters (FDR 0.05 and SD 3), 40 SNPs were related to precipitation seasonality, 38 to annual deficit in water balance, 33 to annual range in temperature, 13 to mean annual temperature, 12 to soil calcium, 9 to mean annual solar radiation, and 3 to mean annual humidity (Table S5).

## 4 Discussion

In this study, we explored the population structure, genetic diversity, evolutionary history, and adaptive potential of *Syzygium maire*, a critically threatened tree endemic to Aotearoa, New Zealand. Our results are based on between 126,386 and 957,330 SNPs identified through low coverage‐whole genome resequencing of 269 wild trees across the species' natural range. We found high levels of differentiation between populations and typical levels of diversity within them compared to other tree species in Aotearoa, including evidence of local‐scale structure. We identified five major clusters across the extant range of *S. maire*: In the North Island: Northland (NOR), Bay of Plenty (BOP) in the central east, Taranaki (TAR) in the central west, and Greater Wellington/Manawatū (GWE/MAN) in the south. A single cluster was identified in the South Island, Marlborough (MAR). All populations, apart from MAR, are genetically diverse. There is evidence of dispersal and divergence, local structure, and admixture, and potential evidence of expansion and then contraction into local refugia, presumably in response to glacial cycling. We also detected genetic variation potentially contributing to range scale climate adaptation, most notably for precipitation seasonality and water deficit. This is the first population genomic study of a tree species dispersed solely by animals in Aotearoa, and the findings of this study will contribute to the restoration and conservation of *S. maire* and swamp forest ecosystems across the country.

Previous work on the flora of Aotearoa has identified broad north–south patterns across species, including evidence for a north‐central‐south split (Heenan et al. 2022; Koot et al. 2022; Chagné et al. 2023; Heenan et al. 2024; McCartney et al. 2024). In most cases, there is also evidence of east–west differentiation. In the case of mānuka ( *Leptospermum scoparium* , Myrtaceae), clusters were similar to those described here, except that TAR and GWE/MAN populations were not separated (Buys et al. 2019; Koot et al. 2022; Chagné et al. 2023). A study on rewarewa (*Knightia excelsa*, Proteaceae) also failed to detect any significant differentiation between TAR and GWE/MAN populations, but showed similar groupings otherwise (McCartney et al. 2024). Taranaki has been suggested to have harbored large, *S. maire* dominant swamplands prior to deforestation (Clarkson 2014), and the separation of TAR and GWE/MAN into separate clusters may reflect more specialist habitat requirements of *S. maire* resulting in landscape‐level segregation of these populations. Separation between the northern, central, and southern populations sampled here does not necessarily imply that these are strictly isolated populations, because most gaps between clusters in the analysis corresponded to areas where samples were not collected. Indeed, while clustering separately on most analyses, the BOP population appears to be an admixed intermediate between other major clusters. Therefore, the population clusters may be less well defined than they appear to be in the current analysis. In this case, more admixed populations would act as intermediaries for genetic exchange. Our analyses partially support this idea, as evidence of admixture appears across all populations. Indeed, movement of seed dispersers would have been facilitated by extensive swamplands interspersed among forest habitats. In mānuka, kānuka, and rewarewa, however, more representative sampling took place across the landscape, and clear genetic clustering was still observed (Heenan et al. 2022, Koot et al. 2022, Chagné et al. 2023, Heenan et al. 2024, McCartney et al. 2024). The same broad‐scale biogeographic boundaries may be responsible for some of the genetic structure we observe in *S. maire*. However, as a habitat specialist with different dispersal mechanisms, *S. maire* may demonstrate unique biogeographic patterns.

We observed relatively high *F*_ST_ compared to other tree species in Aotearoa, much higher than kānuka ( *Kunzea ericoides* , Myrtaceae), but equivalent to rewarewa and pōhutukawa (*Metrosideros excelsa*, Myrtaceae) (Young et al. 2001; Heenan et al. 2022; Chagné et al. 2023; Heenan et al. 2024; McCartney et al. 2024). A recent meta‐analysis for seed plants suggested that pollination syndrome is more important for determining *F*_ST_ than seed dispersal mechanisms, with plants pollinated by small insects in general showing higher *F*_ST_ (Gamba and Muchhala 2020). Here, however, we observe higher *F*_ST_ among trees with mixed pollinators (including birds, bats, and insects, i.e., rewarewa, pōhutukawa, and *S. maire*) than those pollinated primarily by insects (mānuka and kānuka). All apart from *S. maire* also have wind‐dispersed seed, suggesting seed dispersal mechanisms are not the major drivers of these differences. Differences may rather be due to differing habitat specialization or dispersal dynamics. Indeed, low *F*_ST_ observed in kānuka was suggested to be due to a high migration rate. Rewarewa and pōhutukawa, in contrast, exhibit low net migration (Young et al. 2001; McCartney et al. 2024). Mānuka and kānuka are also generalists in their habitat requirements and widely distributed, while rewarewa and pōhutukawa are more specific in their habitat requirements. Higher *F*_ST_ and structure in *S. maire* may therefore be due to lower dispersal ability and niche specialization.

We also observed structure at a subregional scale. In large swamp forest stands, close familial relationships may break down as distance from a parent tree is increased. Higher kinship within stands reflects this pattern. As local structure is still present with outliers filtered, it is more likely to be a result of dispersal‐based isolation than selection. This hypothesis remains to be tested but has important implications for reducing the effects of fragmentation at local scales. As wetland is a naturally patchy and dynamic habitat in Aotearoa (dependent on hydrology in a landscape prone to change), and *S. maire* requires very specific habitat conditions, it is also probable that some small populations are short‐lived or experience very low net migration and more severe genetic isolation. This is likely the case for trees in the west of MAR, some BOP sites, and other naturally highly related, inbred patches. Together with the *F*_ST_ and structure results, this suggests that any attempts to protect the genetic diversity of the species need to focus on reduction in population fragmentation through, for example, introducing corridors for gene flow or actively moving genetic material between fragmented stands of trees.

As this species can self‐fertilize, single founders could successfully colonize favorable habitat patches. The inbred MAR stand likely reflects such an event. These trees occupy a small stream estuary and some patchy habitat in the surrounding catchment. Despite kererū being known to fly tens of kilometers (Powlesland et al. 2011), they are generally highly sedentary, especially after feeding, and exhibit maximal seed dispersal distances of around 1.5 km (Wotton and Kelly 2012). The nearest known trees are over 25 km away, with intervening ocean channels and large hills with deep valleys serving as potential barriers to gene flow. The general state of inbreeding in the South Island populations is more likely an artifact of recent extinction of an older, more diverse South Island population than very recent arrival from the North Island. Indeed, there are large tracts of transformed wetland near to where the more diverse eastern individuals were sampled. High kinship in the eastern populations is probably due to the isolation of the population and derivation from a single or very few founders.

In all cases, MAR appears most related to trees in western GWE and MAN. There is some evidence of a land bridge and resultant movement of species between Taranaki and the South Island (Marra et al. 2009). However, the region at that time would have been far cooler and drier, and by the time conditions became more suitable for *S. maire* colonization, it is likely the bridge would have closed. Indeed, South Island vegetation change is only very recent, with podocarp trees becoming more dominant from 6000 years ago (McGlone et al. 1996; Wilmshurst et al. 2002). Our results suggest initial dispersal of *S. maire* into the South Island from an ancestor of the GWE/MAN populations.

At the opposite end of the species range, Northland hosts greater endemism and genetic diversity among many species than elsewhere in Aotearoa (Wood et al. 2017). We observed the highest diversity and lowest *F*_ST_, inbreeding, and kinship in NOR, suggesting it is the longest persisting *S. maire* population. Alternatively, NOR could also have been colonized from different regions, although the admixture results suggest that this would not have been from more southern populations. Aotearoa experienced widespread landscape and environmental change during the Miocene until present. Much of the current landmass was submerged or still to be formed by volcanism and tectonic uplift (Marra et al. 2009; Wood et al. 2017; Strogen et al. 2023). During this time, the only consistent landmass was that in the far north of the North Island, southern North Island, and northern South Island. Northland also seems to have remained more consistently forested than the rest of Aotearoa (Wright et al. 1995; Mildenhall 2003). Admixture and structure results also indicate that NOR is a unique genetic cluster, suggesting against multiple colonizations from other regions leading to the higher diversity and differentiation. It is therefore likely that Northland is the site of original arrival for the species. After NOR, TAR has the lowest *F*_ST_, inbreeding values, and highest diversity, suggesting the continued existence of large, stable populations in the region.

The observed structure and admixture patterns are reflective of a more complex history than solely north–south expansion as a result of glaciation. Indeed, the hypothesis of northern glacial refugia in Aotearoa has been refuted (Wood et al. 2017). Rather, ecological inversions (i.e., a reversal of the relative abundances of species during opposite phases of glacial cycles) better explain the distribution of most species. Vegetation would recede to localized refugia during periods with adverse environmental conditions and act as a seed source once conditions became suitable for expansion. Specifically, cold dry glacial periods characterized by grassland and refugial beech forest (McGlone and Topping 1983; McGlone et al. 1984; Bussell 1988; Marra et al. 2009) have alternated with short, warm and relatively wet interglacials dominated by broadleaf, podocarp forest and wetlands (Bussell 1988; Marra et al. 2009; Wood et al. 2017). In this case, localized population bottlenecks during recession and introgression restoring some heterozygosity to metapopulations upon expansion would be expected, as opposed to sequential founder effects and clinal reduction in diversity as the species spread southward. The last glacial maximum was approximately 30,000 years ago, with warming accelerating from 25,000 to 18,000 years ago (Suggate and Moar 1970; Newnham et al. 2003; Newnham et al. 2007). Cycles occur approximately every 100,000 years (Lisiecki and Raymo 2005). We observe reasonably high diversity relative to the expectation of a complete extinction in the southern range as recently as the last glacial maximum. Short branch length and increase in new branches in the south in many populations suggest recent population expansions. Much deeper branches and more resolved phylogeny on north‐central regions again suggest longer evolutionary histories and more stable populations. Increasing kinship moving southward may be due to both successive founder events during ancient migration and admixture due to localized and more extreme refuge‐radiation dynamics. Overall, these observations indicate larger or longer persisting populations of *S. maire* in the northern reach of its range, with smaller, southern refugia.

Breakdown of clustering and reduction in most summary statistics in the outlier filtered set of SNPs suggest structure is in part being driven by adaptive alleles. Drivers for the structure in mānuka and kānuka were identified to be largely associated with environmental conditions (Chagné et al. 2023; Heenan et al. 2024). We also demonstrated that at least some of the identified outliers were associated with climatic adaptation. Indeed, biogeographic boundaries have been suggested to be largely responsible for the convergent genetic structure observed in Aotearoa species studies to date (Heenan et al. 2022; Heenan et al. 2024). North to south shift in genetic ancestries may also suggest a cline in genetic variation.

GEA analysis identified SNPs associated with seven climactic and two soil character variables. A large amount of this genetic variation was associated with precipitation seasonality and mean annual water deficit. Precipitation seasonality is defined as the difference in precipitation amount between the wet and the dry seasons. Mean annual water deficit captures the average annual difference between the amount of water plants could use if it were available (potential evapotranspiration) and the amount of water actually available (actual evapotranspiration). Seasonal rainfall patterns strongly influence tree growth and physiology, particularly in tropical and subtropical regions (Borchert 1998). In areas with distinct wet and dry seasons, a range of structural and physiological adaptations have been identified to aid in coping with seasonal drought (Borchert 1998; Baltzer et al. 2009; Baltzer and Davies 2012). Adaptation to variability in seasonal precipitation has been shown for other tree species. For instance, in the widespread conifer *Callitris columellaris* , growth responses to rainfall variability differ between semi‐arid and tropical populations, with strong linear relationships between growth and rainfall amount for semi‐arid populations and weaker, asymmetrical responses for tropical ones (O'Donnell et al. 2021). Likewise, a landscape genomics study on *Quercus rugosa* found that precipitation seasonality was a strong environmental driver of turnover in allele frequencies across the species' range (Martins et al. 2018). It is not surprising that variation in seasonal rainfall might drive adaptation in a species that is not drought‐tolerant and requires waterlogged soils, especially at the germination and establishment stage. Drainage of habitat rapidly results in the death of even mature trees, and negatively affects seedling establishment and survival (Balkwill pers. obs.). This is concerning as it is expected that seasonality will increase globally (Wang et al. 2024). It is unclear how precipitation seasonality will change in Aotearoa, but it is expected that much of the species range will become drier with increased drought pressure, especially in the north (MFE 2018). Humidity is expected to decrease and solar radiation is expected to be impacted only minimally. On a positive note, increased seasonality may reduce pathogen load through alternating high and low infection pressure across seasons (Milici et al. 2020), and reduced humidity and rainfall may reduce myrtle rust infection. Identifying variation able to provide adaptation to drought stress may therefore be important to aid in survival in drier conditions and allow the species to persist outside of the climatic conditions that promote high myrtle rust load. Assessing whether the variation identified here is linked to underlying candidate genes will be valuable for validating our findings and determining potential effects on the climatic adaptability of *S. maire*.

It should be noted that variation in inter‐annual precipitation does not imply severe drought, as Aotearoa already has high rainfall. Furthermore, we ran these analyses on individual rainfall variables (rainfall in the wettest and driest quarters and mean annual rainfall) to ensure that these observations were not a confounding effect of absolute rainfall, but did not identify a strong correlation with genetic variation (data not shown). Additionally, failure to discover variation associated with climatic variables may be artefact of sample collection bias. Future studies should aim to sample from a wider range of populations across the species range.

In a conservation context, our evidence suggests that *S. maire* should be broadly grouped according to five distinct conservation management units. Despite the possibility that population structure is due to a lack of sampling of intermediary populations, remnants are sufficiently different and fragmented that they are likely to be functionally isolated populations. Indeed, many intermediary populations would have been lost in land transformation. These distinctions are therefore relevant in a conservation context. In the case of MAR, restoration would best be assisted through the introduction of material from the lower North Island. For the remaining populations, there should be sufficient diversity within clusters to serve restoration goals. Due to the small, isolated MAN population, movement of genetic material from GWE would also be advisable. Natural fragmentation, and potentially cyclical bottlenecking and radiations suggest that *S. maire* has persisted through similar conditions to those currently observed, and that localized, sub‐regional structure is unlikely to be adaptive. Breakdown of much of the observed patterns when outliers are removed and the GEA analysis, however, indicates that there is broader, regional‐scale adaptive variation present. It is therefore crucial that conservation actions take place promptly to capture the current diversity of the species. Failing prompt action, it is likely that ongoing selfing, inbreeding within stands, and external factors reducing reproductive success will lead to significant degradation of the extant wild genetic resources of *S. maire*. In a restoration context, improving population connectivity, including through artificial migration between stands, will be key to mitigating the effects of fragmentation on the species. Evidence of admixture suggests a natural state where gene flow was common across large pre‐agricultural landscapes. More broad‐scale seed sourcing where low diversity and environmental change threaten populations may therefore mimic the natural dynamics of the species. While variation in phenotypic characteristics has not been assessed, growth rates (personal communication Bruce Clarkson, pers. obs.) and secondary metabolites (unpublished data) have been observed to differ between seedlings from different regions when grown in a common garden. At a regional scale, restoration practitioners should therefore be aware that potential adaptive variation may exist. To support this, exploration of local‐scale structure should be prioritized, as multiple spatial scales need to be considered to inform genetic conservation and restoration (Engelhardt et al. 2014). Furthermore, it is expected that Aotearoa will become warmer and wetter, although much of *S. maire*'s range will experience drier summers (MFE 2018). Sourcing seed more adapted to these conditions may therefore be beneficial to the restoration of the species, especially in the southern half of its range.

In conclusion, we found distinct, regional‐scale genetic clusters for *S. maire*, which in turn exhibit local structure and high levels of admixture. These patterns are likely a result of complex, glaciation‐related ecological inversion leading to local‐scale microrefugia and radiations of the species. We also uncovered evidence that at least some of the variation contained within clusters is adaptive. Restoration and conservation goals would best be served by capturing diversity within these clusters and improving population connectivity to promote the natural dispersal state of the species. Future work should aim to explore potential adaptive traits in the species and better address local‐scale dispersal dynamics necessary for the maintenance of sufficiently large population sizes to prevent negative genetic effects of fragmentation. The genomic dataset generated here provides a strong foundation for identifying evolutionarily significant units, management units, and adaptive population groupings (Funk et al. 2012). Defining such conservation units should be considered a key goal for future research, as it would enable targeted strategies to safeguard the species' evolutionary potential. For instance, this could inform whether conservation should prioritize genome‐wide diversity or focus on maintaining functionally relevant adaptive variation (Kardos et al. 2021). Integrating genomic offset approaches into future work may further support this by highlighting populations that may be most vulnerable to environmental change, enabling proactive and climate‐resilient conservation planning.

## Conflicts of Interest

The authors declare no conflicts of interest.

## Supporting information


**Figure S1:** Density plot for minor allele frequency for 1,914,938 SNPs from 269 individual trees. The frequency of the alternative (less common allele) for all sites is plotted on the *x*‐axis, while the density is presented on the *y*‐axis. The majority of the sites have minor allele frequencies below 0.05, with a median of 0.03. A peak is observed at 0.005, with a secondary peak in frequency at approximately 0.02. A rapid decline and long tail toward 0.5 is then observed, suggesting an abundance of rare alleles in the dataset.

**Figure S2:** Circa plot of SNP, repeat and gene density per chromosome (IW). The outermost plot depicts the frequency of SNPs unfiltered for minor allele frequency (MAF0.00), followed by SNPs filtered for a MAF of 0.05, repeat density and gene density. All statistics were calculated on 250,000 bp windows. *Y*‐axis limits for each track are shown in the top left corner of each plot of chromosome two. Circos plots were created in Circa (http://omgenomics.com/circa).

**Figure S3:** Linkage disequilibrium (LD) decay as a function of genomic distance for each of 11 chromosomes and four different levels of minor allele filtering. LD was calculated on 1000 bp windows with all alleles retained and expressed as the squared correlation coefficient (*r* ^2^).

**Figure S4:** Screeplots representing the contribution of eigenvalues (PC) to the proportion of explained variance for various filtering parameters. Plots were generated in order to determine the appropriate cutoff for outlier detection. Cutoffs according to Cattell's rule (Cattell 1966) were not obvious but in all cases it appeared that the majority of variance was explained with between 3 and 6 PCs. The first plot did not have any minor allele frequency filtering applied while the following two datasets were filtered for a frequency of 0.05. The first and last plots were filtered for linkage disequilibrium. Cattell's rule specifies that eigenvalues contributing to non‐random variation fall to the left of the straight line (Cattell 1966).

**Figure S5:** Principal component analysis (PCA) for 269 S. maire trees sampled across Aotearoa with varying levels of minor allele frequency (MAF) and linkage disequilibrium (LD) filtering. The number of retained SNPs for each filtering combination is shown in the bottom right corner of the graph space of each scatter plot. Each point depicts a single sample. Individuals are coloured according to subregion from which they were sampled.

**Figure S6:** Principal component analysis (PCA) for 269 S. maire trees sampled across Aotearoa with minor allele frequency (MAF) of 0.05, filtered for linkage disequilibrium (LD) and outlier alleles. Analysis was performed on 126,386 SNPs. Each point depicts a single sample. Individuals are coloured according to subregion from which they were sampled.

**Figure S7:** ADMIXTURE analysis for 203 individual trees sampled across Aotearoa and filtered for outliers. To generate the plot, ADMIXTURE was run for a dataset of 138,267 SNPs filtered for a minor allele frequency of 0.05, linkage disequilibrium, related individuals and outliers. The outcome is an estimation of the proportion of ancestry for each individual derived from a number of hypothetical ancestral populations (as defined by K). Individuals are organized according to region, roughly from north at the left of the plot to southern populations at the right. We assessed the shared ancestry for two to seven common ancestors, with each colour and the proportion of the bar graph it occupies corresponding to an ancestral population and its contribution to that individual. The barcode above the plot indicates the subpopulations from which individuals are derived, as per the legend. BOP, Bay of Plenty; GWE, Greater Wellington; MAN, Manawatū; MAR, Marlborough; NOR, Northland; TAR, Taranaki.

**Figure S8:** NeighbourNet analysis depicted as a phylogenetic network for 269 individual S. maire trees. Pairwise Nei's D for all pairs of individuals calculated on 188,131 SNPs filtered for linkage disequilibrium and minor allele frequency of 0.05 was used as input for in SplitsTree v6.1.16 (Huson 1998). Phylogenetic uncertainty is depicted by edges showing connections at more than a single point. Each terminal edge represents an individual. Groups of individuals are coloured according to the region from which they were sampled.

**Figure S9:** Neighbour joining analysis for 269 individual S. maire trees. Pairwise Nei's D for all pairs of individuals calculated on 188,131 SNPs with filtering for linkage disequilibrium and minor allele frequency of 0.05 was used as input for SplitsTree v6.1.16 (Huson 1998). Colours depict broad geographic regions. BOP, Bay of Plenty; GWE, Greater Wellington; MAN, Manawatū; MAR, Marlborough; NOR, Northland; TAR, Taranaki. The figure was created in FigTree v1.4.4.

**Figure S10:** Gene diversity (heterozygosity) for each sampling region with outlier alleles removed. Statistics were calculated on 126,386 SNPs filtered for linkage disequilibrium, minor allele frequency of 0.05 and outlier alleles. BET, between population diversity; BOP, Bay of Plenty; GWE, Greater Wellington; MAN, Manawatū; MAR, Marlborough; NOR, Northland; TAR, Taranaki. The mean values for each region are denoted by (+).

**Figure S11:** Inbreeding coefficients for individuals grouped according to region. BOP, Bay of Plenty; GWE, Greater Wellington; MAN, Manawatū; MAR, Marlborough; NOR, Northland; TAR, Taranaki.

**Figure S12:** Pairwise correlation and data distribution for 10 environmental variables. Pairwise comparisons and line of best for each datapoint are depicted below the diagonal. Pearson's correlation coefficient between environmental variables is presented above the diagonal. Histograms of data distribution for each environmental variable are shown on the diagonal.

**Figure S13:** The contribution to variation for each RDA axis. Plots were generated in order to determine the appropriate number of eigenvalues for RDA analysis.

**Figure S14:** Manhattan plot of LFMM analysis for various environmental variables. Significance of SNP association (−1og10 of the *q*‐value) with each respective environmental variable is show on the *y*‐axis. Chromosomes and cumulative position of SNPs is show on the *y*‐axis. A significance cut‐off of 0.05 (red dashed line) was chosen. SNPs depicted in red were also identified by the RDA analysis associated with that environmental variable and present in the outliers detected by pcadapt.

**Figure S15:** Manhattan plot of LFMM analysis for various environmental variables. Significance of SNP association (−1og10 of the *q*‐value) with each respective environmental variable is show on the *y*‐axis. Chromosomes and cumulative position of SNPs is show on the *y*‐axis. A significance cut‐off of 0.05 (red dashed line) was chosen. SNPs depicted in red were also identified by the RDA analysis associated with that environmental variable and present in the outliers detected by pcadapt.

**Table S1:** Breakdown of samples by regions, subregions, genetic cluster and sample size for the 269 trees characterized in this study.

**Table S2:** Variant calling statistics for single nucleotide polymorphisms (SNPs) according to: varying minor allele frequency (MAF), linkage disequilibrium (LD) and outlier filters.

**Table S3:** Pairwise *F*_ST_ and confidence intervals for two datasets, one with and one without outlier alleles.

**Table S4:** Partial redundancy analysis (pRDA) accounting for the effect of climate and neutral genetic structure.

**Table S5:** Number of SNPs associated with various environmental variables for less (*q* value < 0.05 and SD 2.5) and more (*q*‐value < 0.1 and SD 3) stringent threshold filters for LFMM and RDA analysis respectively, and overlap with SNPs identified as PCA outliers.

## Data Availability

Raw fastq files and filtered vcf are available from the Aotearoa Genomic Data Repository (Te Aika et al. 2023) at https://data.agdr.org.nz/ (https://doi.org/10.57748/1q9v‐pr20) with permission access to be consented from mana whenua for each specific dataset where it was requested to do so.
