# Supplementary material for: Adaptive Potential of Syzygium maire, a Critically Threatened Habitat Specialist Tree Species in Aotearoa New Zealand
Source: Evol Appl. 2025 Oct 2;18(10):e70161. doi: 10.1111/eva.70161 (PMC12489745; doi:10.1111/eva.70161)
Supplement: Supplementary file 1 — Figure S1: Density plot for minor allele frequency for 1,914,938 SNPs from 269 individual trees. The frequency of the alternative (less common allele) for all sites is plotted on the x‐axis, while the density is presented on the y‐axis. The majority of the sites have minor allele frequencies below 0.05, with a median of 0.03. A peak is observed at 0.005, with a secondary peak in frequency at approximately 0.02. A rapid decline and long tail toward 0.5 is then observed, suggesting an abundance of rare alleles in the dataset. [file EVA-18-e70161-s007.docx]

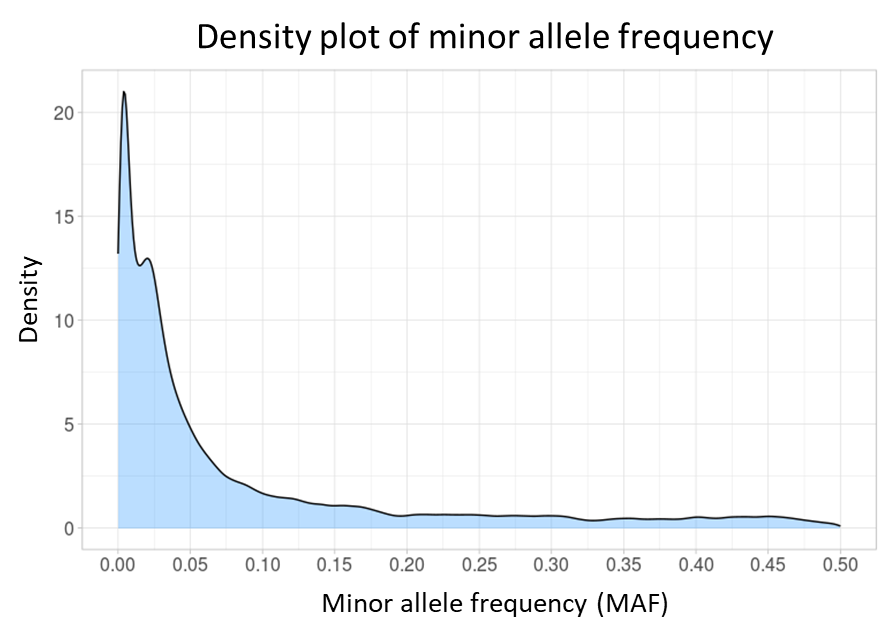


**Figure S1:** **Density plot for minor allele frequency for 1,914,938 SNPs from 269 individual trees.** The frequency of the alternative (less common allele) for all sites is plotted on the x-axis, while the density is presented on the y-axis. The majority of the sites have minor allele frequencies below 0.05, with a median of 0.03. A peak is observed at 0.005, with a secondary peak in frequency at approximately 0.02. A rapid decline and long tail toward 0.5 is then observed, suggesting an abundance of rare alleles in the dataset.
