# Supplementary material for: Adaptive Potential of Syzygium maire, a Critically Threatened Habitat Specialist Tree Species in Aotearoa New Zealand
Source: Evol Appl. 2025 Oct 2;18(10):e70161. doi: 10.1111/eva.70161 (PMC12489745; doi:10.1111/eva.70161)
Supplement: Supplementary file 2 — Figure S2: Circa plot of SNP, repeat and gene density per chromosome (IW). The outermost plot depicts the frequency of SNPs unfiltered for minor allele frequency (MAF0.00), followed by SNPs filtered for a MAF of 0.05, repeat density and gene density. All statistics were calculated on 250,000 bp windows. Y‐axis limits for each track are shown in the top left corner of each plot of chromosome two. Circos plots were created in Circa (http://omgenomics.com/circa). [file EVA-18-e70161-s020.docx]

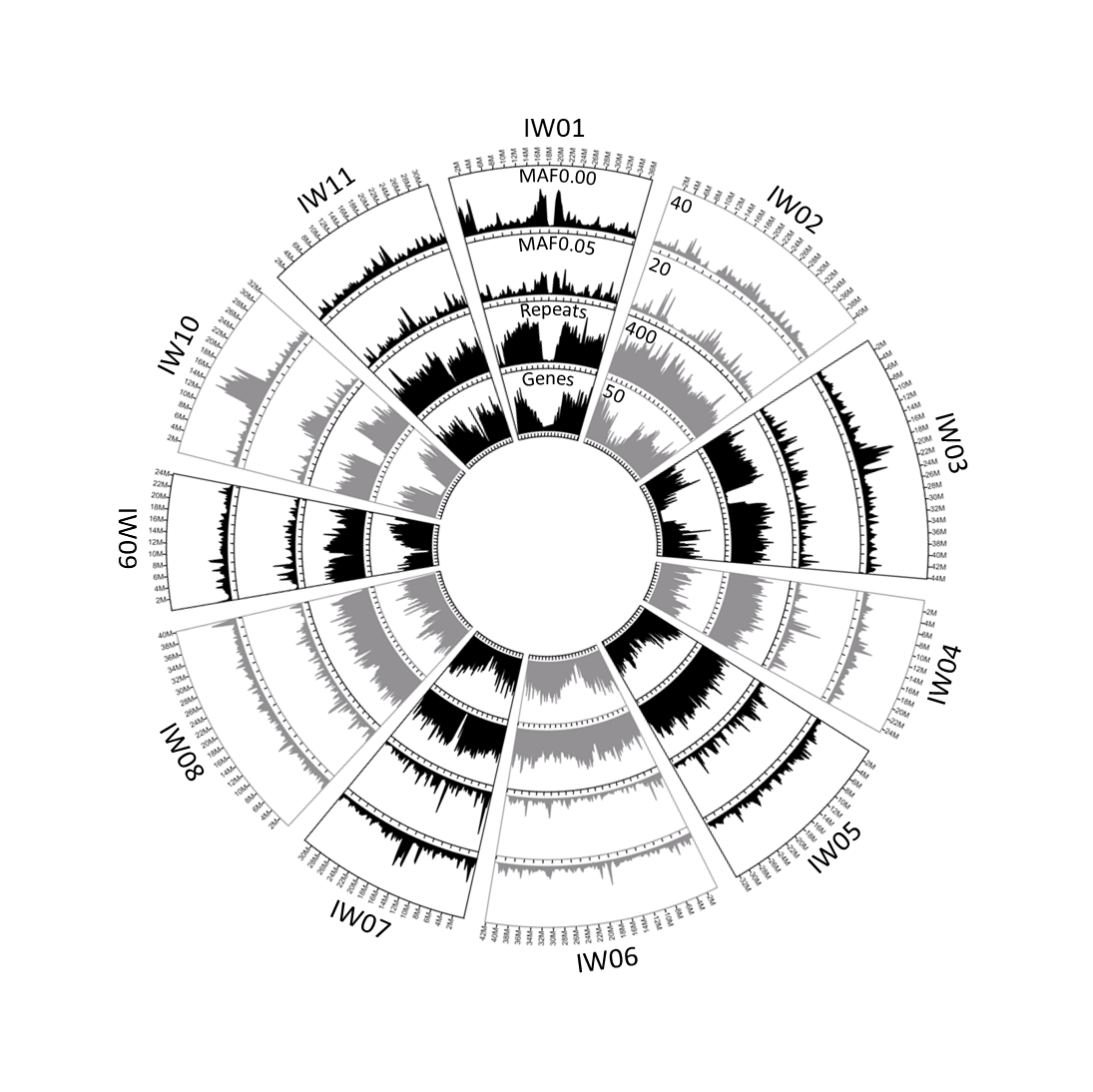


**Figure S2:** **Circa plot of SNP, repeat and gene density per chromosome (IW).** The outermost plot depicts the frequency of SNPs unfiltered for minor allele frequency (MAF0.00), followed by SNPs filtered for a MAF of 0.05, repeat density and gene density. All statistics were calculated on 250,000bp windows. Y-axis limits for each track are shown in the top left corner of each plot of chromosome two. Circos plots were created in Circa (<http://omgenomics.com/circa>).
