# Supplementary material for: Adaptive Potential of Syzygium maire, a Critically Threatened Habitat Specialist Tree Species in Aotearoa New Zealand
Source: Evol Appl. 2025 Oct 2;18(10):e70161. doi: 10.1111/eva.70161 (PMC12489745; doi:10.1111/eva.70161)
Supplement: Supplementary file 3 — Figure S3: Linkage disequilibrium (LD) decay as a function of genomic distance for each of 11 chromosomes and four different levels of minor allele filtering. LD was calculated on 1000 bp windows with all alleles retained and expressed as the squared correlation coefficient (r 2). [file EVA-18-e70161-s002.docx]

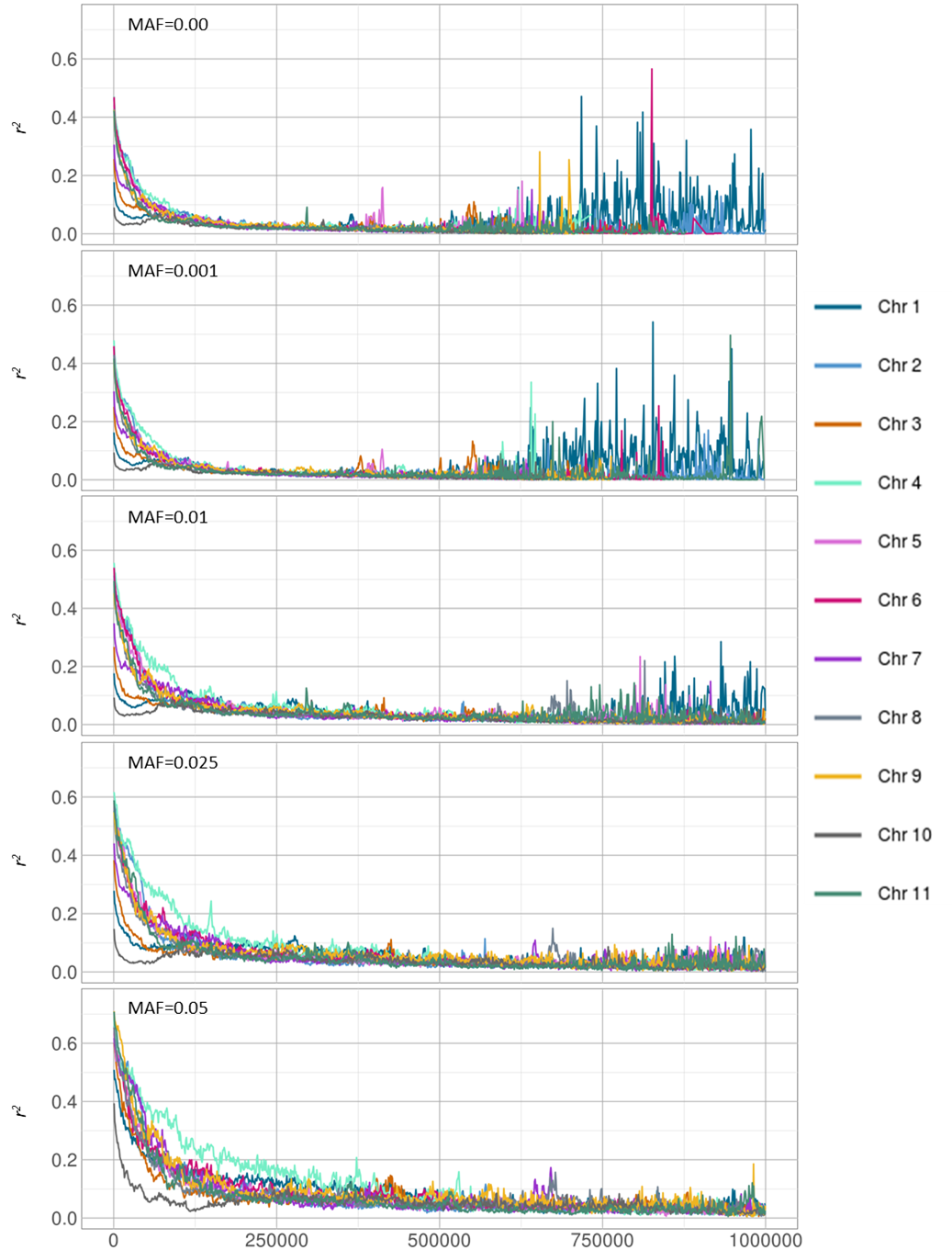


**Figure S3: Linkage disequilibrium (LD) decay as a function of genomic distance for each of 11 chromosomes and four different levels of minor allele filtering.** LD was calculated on 1000bp windows with all alleles retained and expressed as the squared correlation coefficient (r^2^).
