# Supplementary material for: Adaptive Potential of Syzygium maire, a Critically Threatened Habitat Specialist Tree Species in Aotearoa New Zealand
Source: Evol Appl. 2025 Oct 2;18(10):e70161. doi: 10.1111/eva.70161 (PMC12489745; doi:10.1111/eva.70161)
Supplement: Supplementary file 4 — Figure S4: Screeplots representing the contribution of eigenvalues (PC) to the proportion of explained variance for various filtering parameters. Plots were generated in order to determine the appropriate cutoff for outlier detection. Cutoffs according to Cattell's rule (Cattell 1966) were not obvious but in all cases it appeared that the majority of variance was explained with between 3 and 6 PCs. The first plot did not have any minor allele frequency filtering applied while the following two datasets were filtered for a frequency of 0.05. The first and last plots were filtered for linkage disequilibrium. Cattell's rule specifies that eigenvalues contributing to non‐random variation fall to the left of the straight line (Cattell 1966). [file EVA-18-e70161-s011.docx]

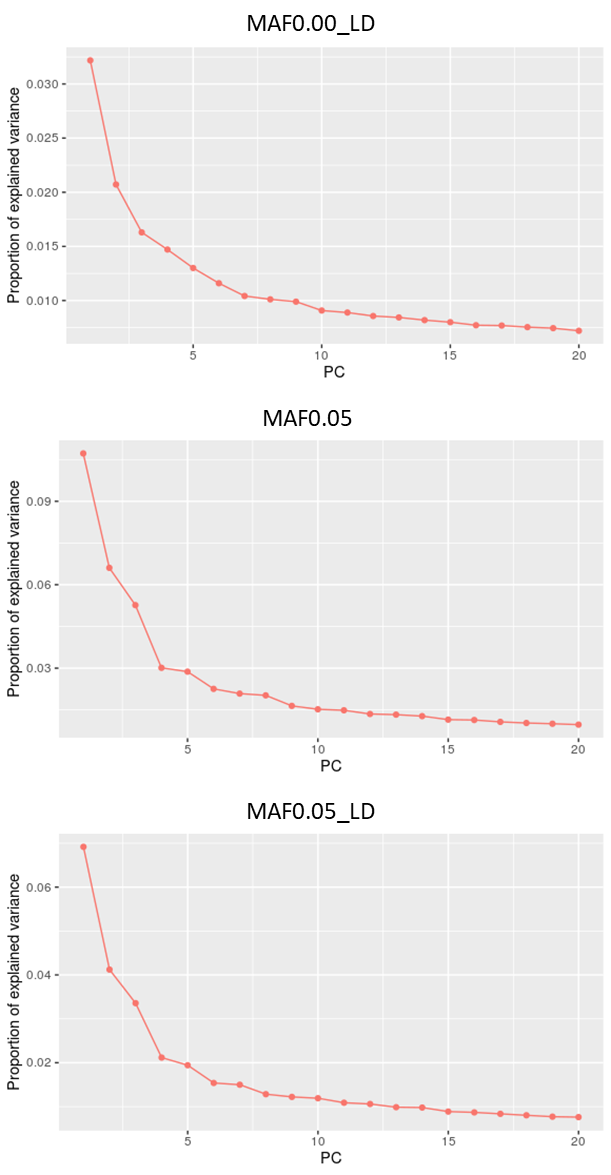


**Figure S4:** **Screeplots representing the contribution of eigenvalues (PC) to the proportion of explained variance for various filtering parameters.** Plots were generated in order to determine the appropriate cutoff for outlier detection. Cutoffs according to Cattell’s rule (Cattell 1966) were not obvious but in all cases it appeared that the majority of variance was explained with between 3 and 6 PCs. The first plot did not have any minor allele frequency filtering applied while the following two datasets were filtered for a frequency of 0.05. The first and last plots were filtered for linkage disequilibrium. Cattell’s rule specifies that eigenvalues contributing to non-random variation fall to the left of the straight line (Cattell 1966).
