# Supplementary material for: Adaptive Potential of Syzygium maire, a Critically Threatened Habitat Specialist Tree Species in Aotearoa New Zealand
Source: Evol Appl. 2025 Oct 2;18(10):e70161. doi: 10.1111/eva.70161 (PMC12489745; doi:10.1111/eva.70161)
Supplement: Supplementary file 5 — Figure S5: Principal component analysis (PCA) for 269 S. maire trees sampled across Aotearoa with varying levels of minor allele frequency (MAF) and linkage disequilibrium (LD) filtering. The number of retained SNPs for each filtering combination is shown in the bottom right corner of the graph space of each scatter plot. Each point depicts a single sample. Individuals are coloured according to subregion from which they were sampled. [file EVA-18-e70161-s008.docx]

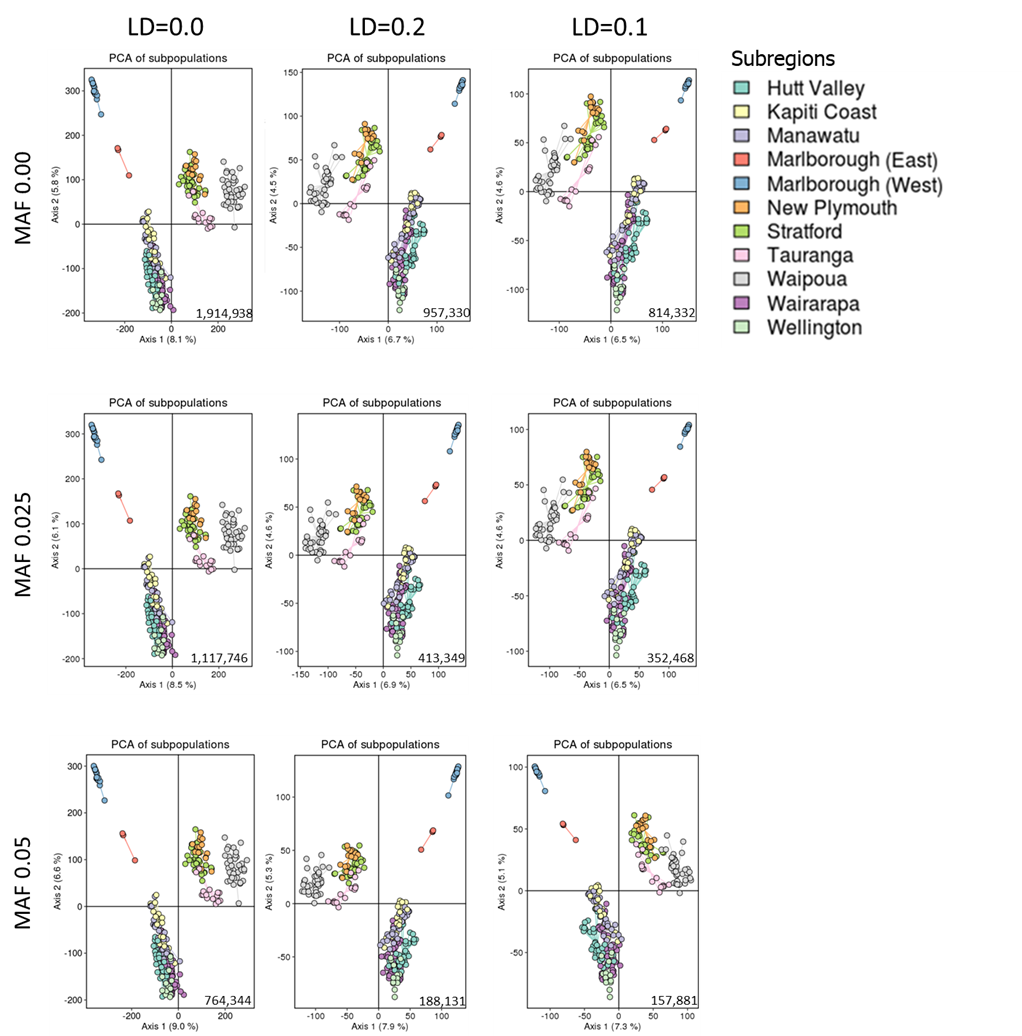


**Figure S5:** **Principal component analysis (PCA) for 269 S. maire trees sampled across Aotearoa with varying levels of minor allele frequency (MAF) and linkage disequilibrium (LD) filtering.** The number of retained SNPs for each filtering combination is shown in the bottom right corner of the graph space of each scatter plot. Each point depicts a single sample. Individuals are coloured according to subregion from which they were sampled.
