# Supplementary material for: Adaptive Potential of Syzygium maire, a Critically Threatened Habitat Specialist Tree Species in Aotearoa New Zealand
Source: Evol Appl. 2025 Oct 2;18(10):e70161. doi: 10.1111/eva.70161 (PMC12489745; doi:10.1111/eva.70161)
Supplement: Supplementary file 6 — Figure S6: Principal component analysis (PCA) for 269 S. maire trees sampled across Aotearoa with minor allele frequency (MAF) of 0.05, filtered for linkage disequilibrium (LD) and outlier alleles. Analysis was performed on 126,386 SNPs. Each point depicts a single sample. Individuals are coloured according to subregion from which they were sampled. [file EVA-18-e70161-s019.docx]

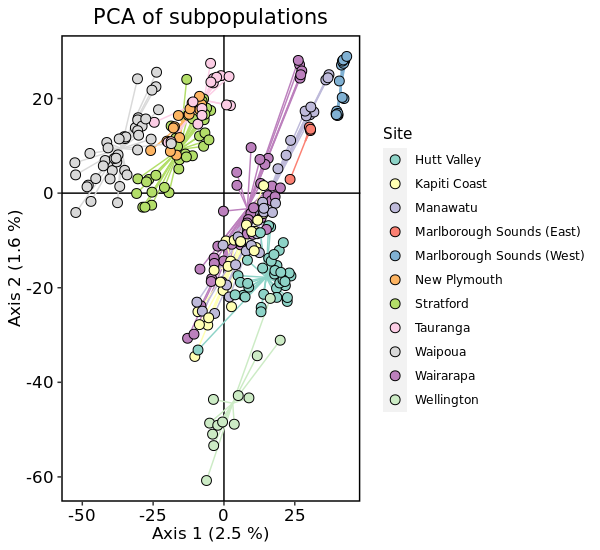


**Figure S6: Principal component analysis (PCA) for 269 S. maire trees sampled across Aotearoa with minor allele frequency (MAF) of 0.05, filtered for linkage disequilibrium (LD) and outlier alleles.** Analysis was performed on 126,386 SNPs. Each point depicts a single sample. Individuals are coloured according to subregion from which they were sampled.
