# Supplementary material for: Adaptive Potential of Syzygium maire, a Critically Threatened Habitat Specialist Tree Species in Aotearoa New Zealand
Source: Evol Appl. 2025 Oct 2;18(10):e70161. doi: 10.1111/eva.70161 (PMC12489745; doi:10.1111/eva.70161)
Supplement: Supplementary file 7 — Figure S7: ADMIXTURE analysis for 203 individual trees sampled across Aotearoa and filtered for outliers. To generate the plot, ADMIXTURE was run for a dataset of 138,267 SNPs filtered for a minor allele frequency of 0.05, linkage disequilibrium, related individuals and outliers. The outcome is an estimation of the proportion of ancestry for each individual derived from a number of hypothetical ancestral populations (as defined by K). Individuals are organized according to region, roughly from north at the left of the plot to southern populations at the right. We assessed the shared ancestry for two to seven common ancestors, with each colour and the proportion of the bar graph it occupies corresponding to an ancestral population and its contribution to that individual. The barcode above the plot indicates the subpopulations from which individuals are derived, as per the legend. BOP, Bay of Plenty; GWE, Greater Wellington; MAN, Manawatū; MAR, Marlborough; NOR, Northland; TAR, Taranaki. [file EVA-18-e70161-s004.docx]

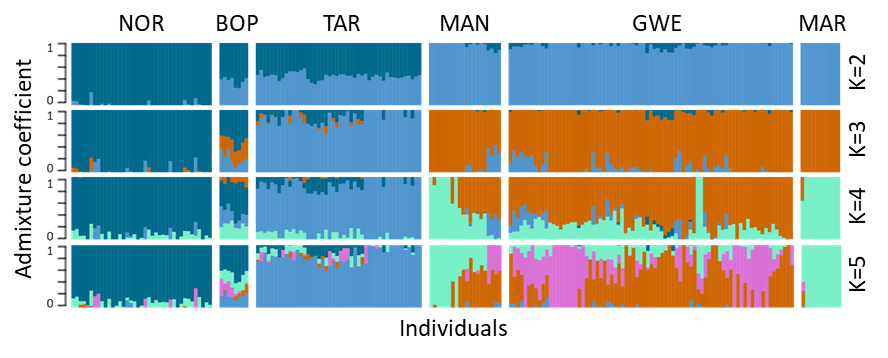


**Figure S7:** **ADMIXTURE analysis for 203 individual trees sampled across Aotearoa and filtered for outliers.** To generate the plot, ADMIXTURE was run for a dataset of 138,267 SNPs filtered for a minor allele frequency of 0.05, linkage disequilibrium, related individuals and outliers. The outcome is an estimation of the proportion of ancestry for each individual derived from a number of hypothetical ancestral populations (as defined by K). Individuals are organised according to region, roughly from north at the left of the plot to southern populations at the right. We assessed the shared ancestry for two to seven common ancestors, with each colour and the proportion of the bar graph it occupies corresponding to an ancestral population and its contribution to that individual. The barcode above the plot indicates the subpopulations from which individuals are derived, as per the legend. Abbreviations for regions are: Northland (NOR), Bay of Plenty (BOP), Taranaki (TAR), Manawatū (MAN), Greater Wellington (GWE), Marlborough (MAR).
