# Supplementary material for: Adaptive Potential of Syzygium maire, a Critically Threatened Habitat Specialist Tree Species in Aotearoa New Zealand
Source: Evol Appl. 2025 Oct 2;18(10):e70161. doi: 10.1111/eva.70161 (PMC12489745; doi:10.1111/eva.70161)
Supplement: Supplementary file 8 — Figure S8: NeighbourNet analysis depicted as a phylogenetic network for 269 individual S. maire trees. Pairwise Nei's D for all pairs of individuals calculated on 188,131 SNPs filtered for linkage disequilibrium and minor allele frequency of 0.05 was used as input for in SplitsTree v6.1.16 (Huson 1998). Phylogenetic uncertainty is depicted by edges showing connections at more than a single point. Each terminal edge represents an individual. Groups of individuals are coloured according to the region from which they were sampled. [file EVA-18-e70161-s018.docx]

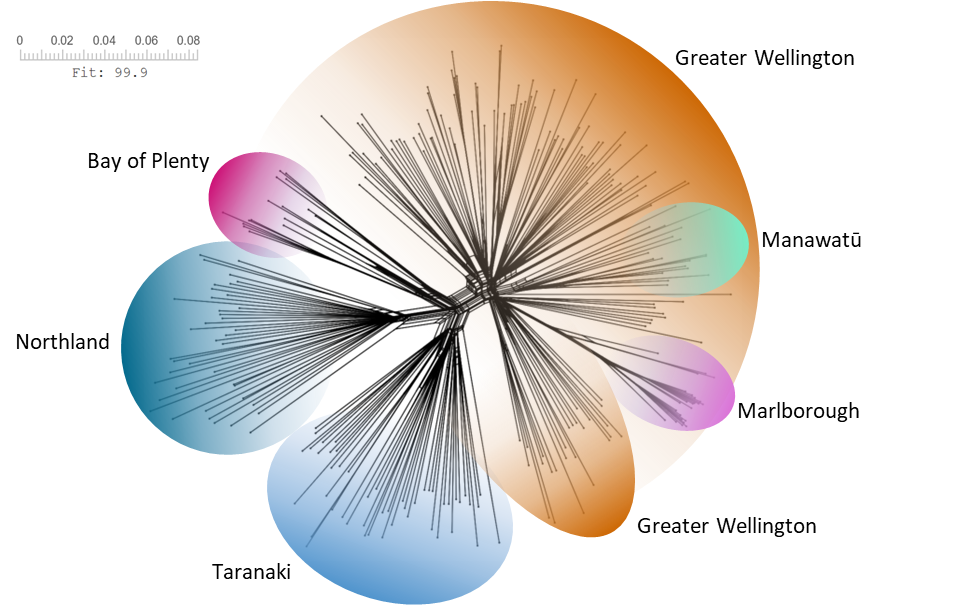


**Figure S8: NeighbourNet analysis depicted as a phylogenetic network for 269 individual S. maire trees.** Pairwise Nei’s D for all pairs of individuals calculated on 188,131 SNPs filtered for linkage disequilibrium and minor allele frequency of 0.05 was used as input for in SplitsTree v6.1.16 (Huson 1998). Phylogenetic uncertainty is depicted by edges showing connections at more than a single point. Each terminal edge represents an individual. Groups of individuals are coloured according to the region from which they were sampled.
