# Supplementary material for: Adaptive Potential of Syzygium maire, a Critically Threatened Habitat Specialist Tree Species in Aotearoa New Zealand
Source: Evol Appl. 2025 Oct 2;18(10):e70161. doi: 10.1111/eva.70161 (PMC12489745; doi:10.1111/eva.70161)
Supplement: Supplementary file 9 — Figure S9: Neighbour joining analysis for 269 individual S. maire trees. Pairwise Nei's D for all pairs of individuals calculated on 188,131 SNPs with filtering for linkage disequilibrium and minor allele frequency of 0.05 was used as input for SplitsTree v6.1.16 (Huson 1998). Colours depict broad geographic regions. BOP, Bay of Plenty; GWE, Greater Wellington; MAN, Manawatū; MAR, Marlborough; NOR, Northland; TAR, Taranaki. The figure was created in FigTree v1.4.4. [file EVA-18-e70161-s006.docx]

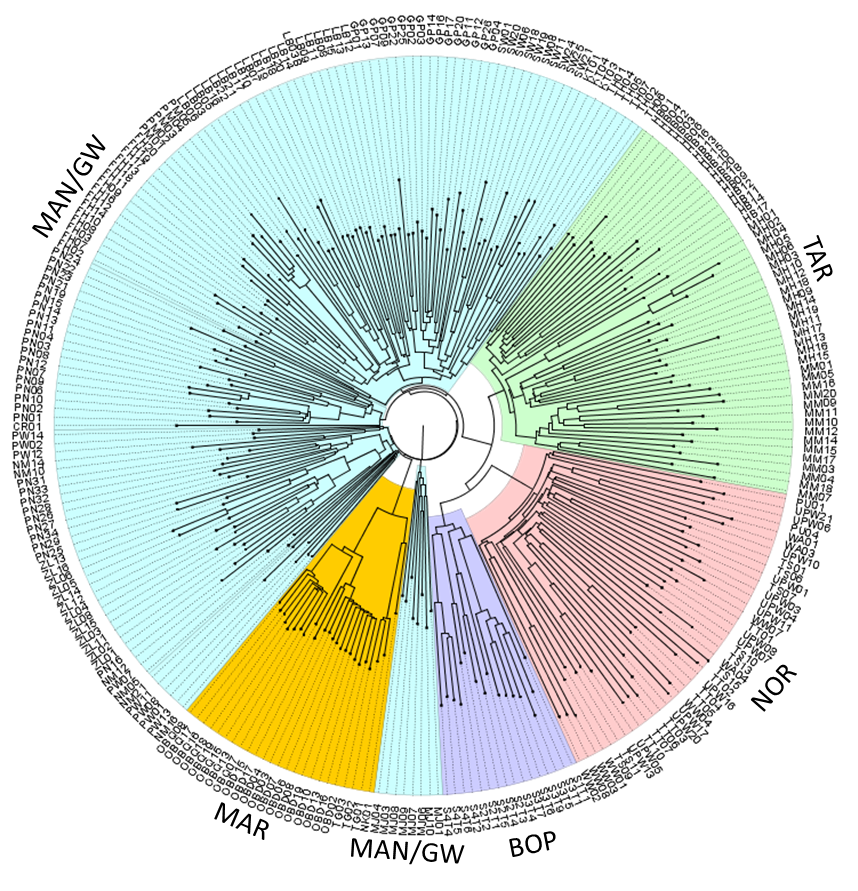


**Figure S9:** **Neighbour joining analysis for 269 individual S. maire trees.** Pairwise Nei’s D for all pairs of individuals calculated on 188,131 SNPs with filtering for linkage disequilibrium and minor allele frequency of 0.05 was used as input for SplitsTree v6.1.16 (Huson 1998). Colours depict broad geographic regions. Abbreviations per region are: Northland (NOR), Bay of Plenty (BOP), Taranaki (TAR), Manawatū (MAN), Greater Wellington (GWE) and Marlborough (MAR). The figure was created in FigTree v1.4.4.
