# Supplementary material for: Adaptive Potential of Syzygium maire, a Critically Threatened Habitat Specialist Tree Species in Aotearoa New Zealand
Source: Evol Appl. 2025 Oct 2;18(10):e70161. doi: 10.1111/eva.70161 (PMC12489745; doi:10.1111/eva.70161)
Supplement: Supplementary file 10 — Figure S10: Gene diversity (heterozygosity) for each sampling region with outlier alleles removed. Statistics were calculated on 126,386 SNPs filtered for linkage disequilibrium, minor allele frequency of 0.05 and outlier alleles. BET, between population diversity; BOP, Bay of Plenty; GWE, Greater Wellington; MAN, Manawatū; MAR, Marlborough; NOR, Northland; TAR, Taranaki. The mean values for each region are denoted by (+). [file EVA-18-e70161-s016.docx]

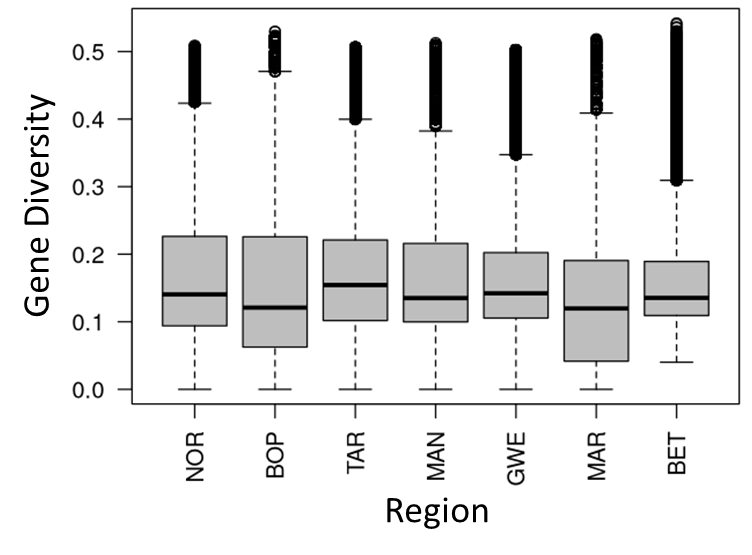


**Figure S10:** **Gene diversity (heterozygosity) for each sampling region with outlier alleles removed.** Statistics were calculated on 126,386 SNPs filtered for linkage disequilibrium, minor allele frequency of 0.05 and outlier alleles. Abbreviations per region are: Northland (NOR), Bay of Plenty (BOP), Taranaki (TAR), Manawatū (MAN), Greater Wellington (GWE), Marlborough (MAR) and between population diversity (BET). The mean values for each region are denoted by (+).
