# Supplementary material for: Adaptive Potential of Syzygium maire, a Critically Threatened Habitat Specialist Tree Species in Aotearoa New Zealand
Source: Evol Appl. 2025 Oct 2;18(10):e70161. doi: 10.1111/eva.70161 (PMC12489745; doi:10.1111/eva.70161)
Supplement: Supplementary file 11 — Figure S11: Inbreeding coefficients for individuals grouped according to region. BOP, Bay of Plenty; GWE, Greater Wellington; MAN, Manawatū; MAR, Marlborough; NOR, Northland; TAR, Taranaki. [file EVA-18-e70161-s010.docx]

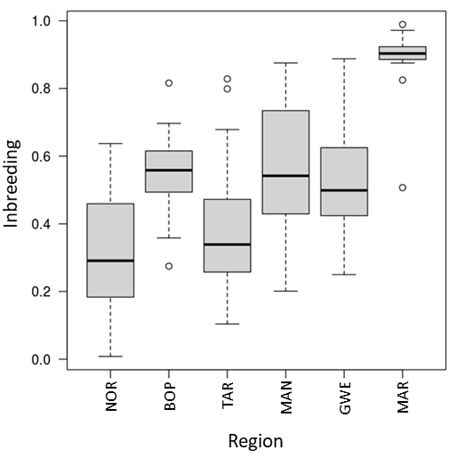


**Figure S11: Inbreeding coefficients for individuals grouped according to region.** Abbreviations per region are: Northland (NOR), Bay of Plenty (BOP), Taranaki (TAR), Manawatū (MAN), Greater Wellington (GWE) and Marlborough (MAR).
