# Supplementary material for: Adaptive Potential of Syzygium maire, a Critically Threatened Habitat Specialist Tree Species in Aotearoa New Zealand
Source: Evol Appl. 2025 Oct 2;18(10):e70161. doi: 10.1111/eva.70161 (PMC12489745; doi:10.1111/eva.70161)
Supplement: Supplementary file 12 — Figure S12: Pairwise correlation and data distribution for 10 environmental variables. Pairwise comparisons and line of best for each datapoint are depicted below the diagonal. Pearson's correlation coefficient between environmental variables is presented above the diagonal. Histograms of data distribution for each environmental variable are shown on the diagonal. [file EVA-18-e70161-s001.docx]

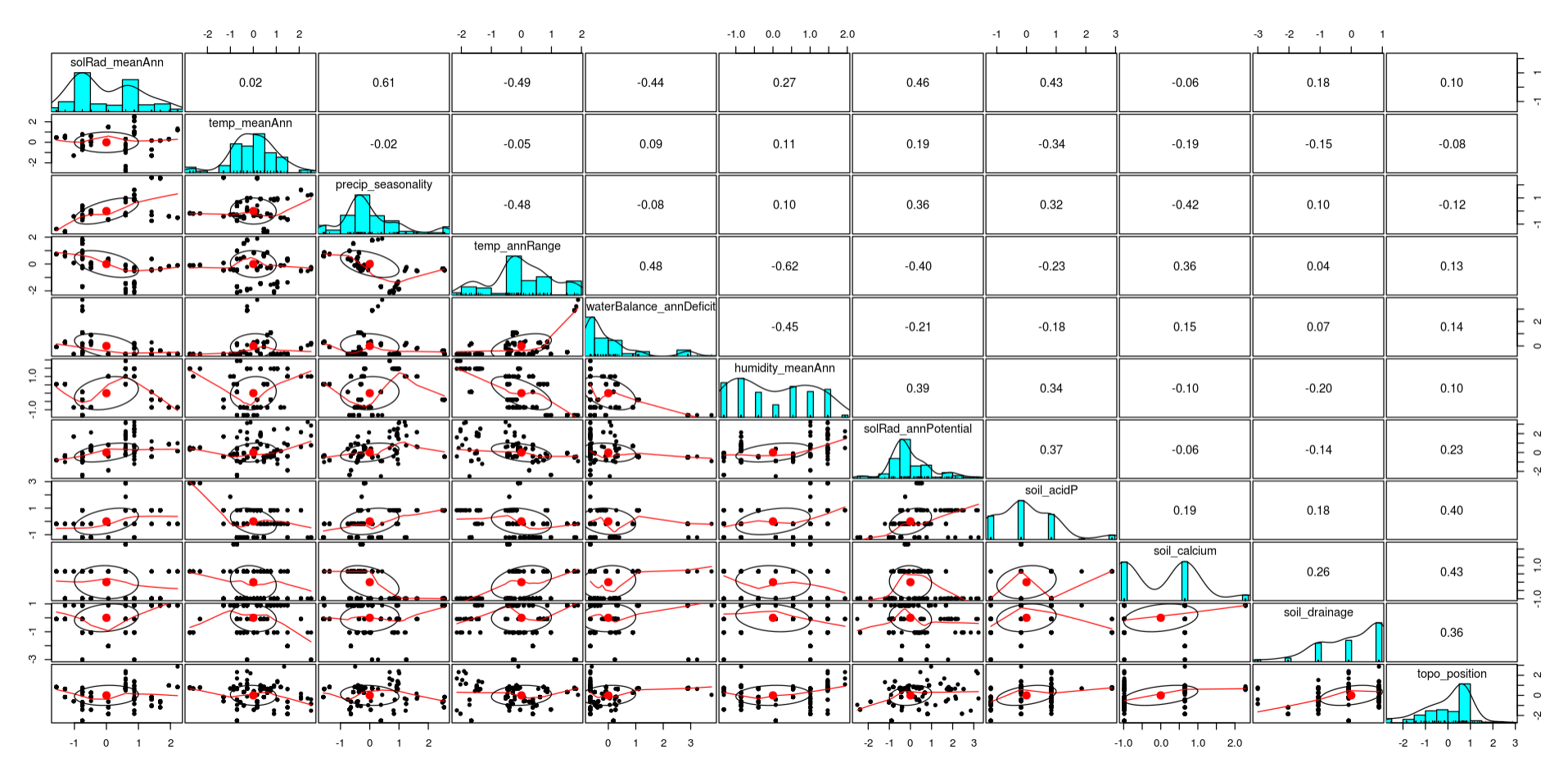


**Figure S12: Pairwise correlation and data distribution for 10 environmental variables.** Pairwise comparisons and line of best for each datapoint are depicted below the diagonal. Pearson’s correlation coefficient between environmental variables is presented above the diagonal. Histograms of data distribution for each environmental variable are shown on the diagonal.
