# Supplementary material for: Adaptive Potential of Syzygium maire, a Critically Threatened Habitat Specialist Tree Species in Aotearoa New Zealand
Source: Evol Appl. 2025 Oct 2;18(10):e70161. doi: 10.1111/eva.70161 (PMC12489745; doi:10.1111/eva.70161)
Supplement: Supplementary file 13 — Figure S13: The contribution to variation for each RDA axis. Plots were generated in order to determine the appropriate number of eigenvalues for RDA analysis. [file EVA-18-e70161-s005.docx]

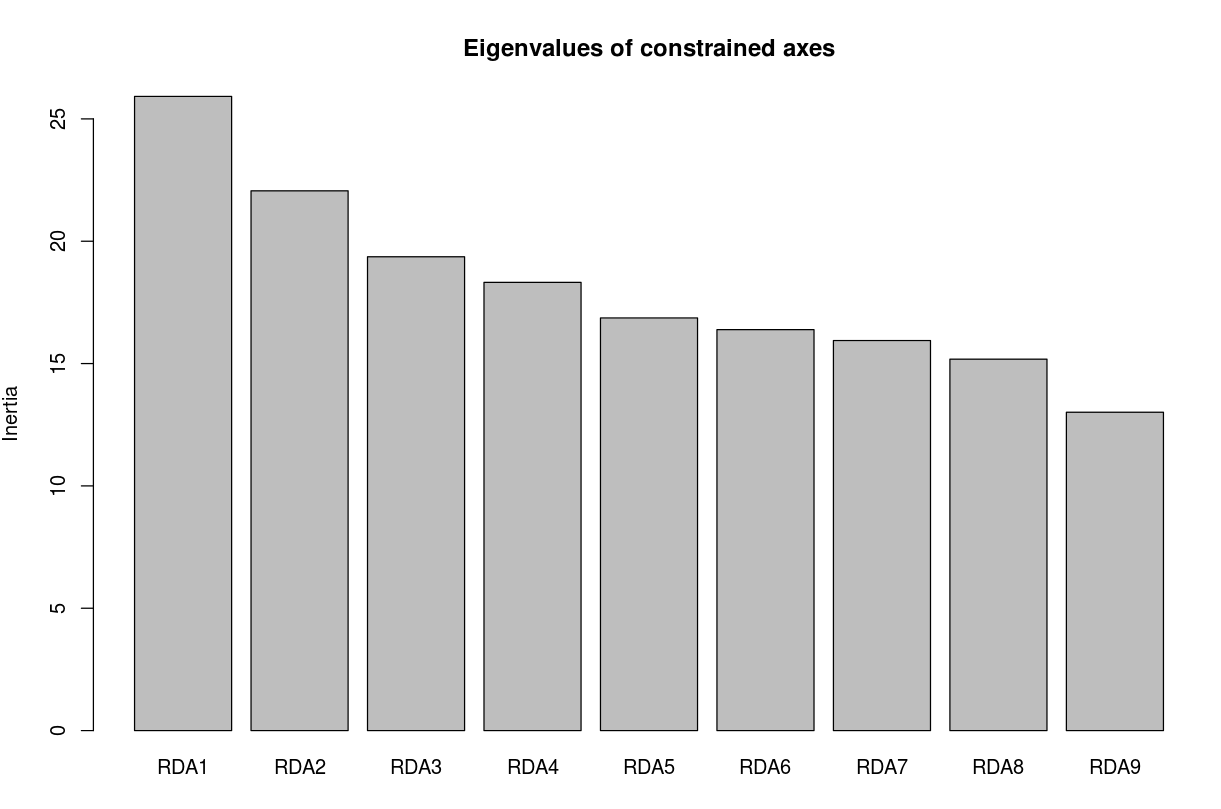


**Figure S13: The contribution to variation for each RDA axis.** Plots were generated in order to determine the appropriate number of eigenvalues for RDA analysis.
