# Supplementary material for: Adaptive Potential of Syzygium maire, a Critically Threatened Habitat Specialist Tree Species in Aotearoa New Zealand
Source: Evol Appl. 2025 Oct 2;18(10):e70161. doi: 10.1111/eva.70161 (PMC12489745; doi:10.1111/eva.70161)
Supplement: Supplementary file 14 — Figure S14: Manhattan plot of LFMM analysis for various environmental variables. Significance of SNP association (−1og10 of the q‐value) with each respective environmental variable is show on the y‐axis. Chromosomes and cumulative position of SNPs is show on the y‐axis. A significance cut‐off of 0.05 (red dashed line) was chosen. SNPs depicted in red were also identified by the RDA analysis associated with that environmental variable and present in the outliers detected by pcadapt. [file EVA-18-e70161-s012.docx]

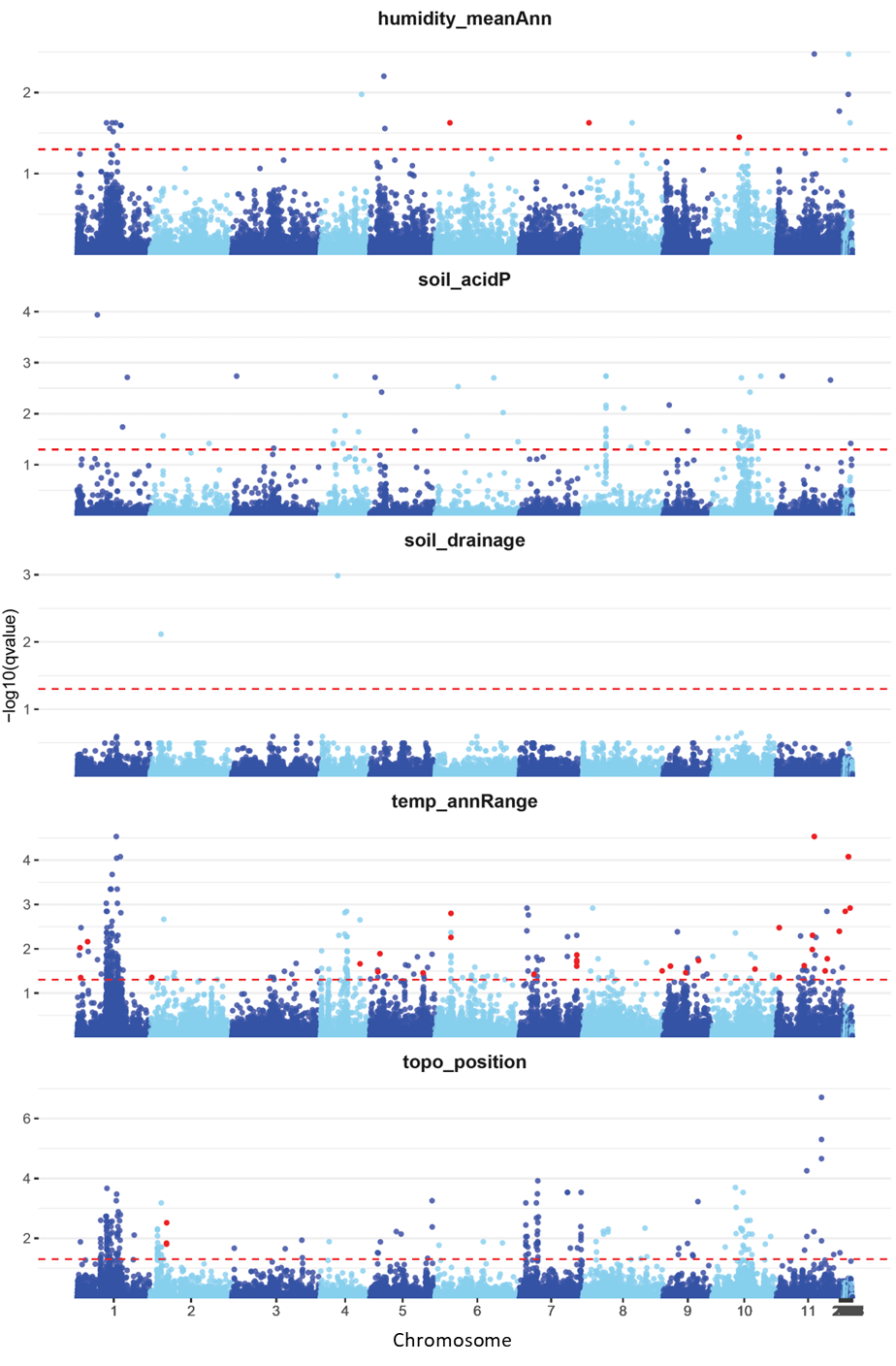


**Figure S14:** **Manhattan plot of LFMM analysis for various environmental variables.** Significance of SNP association (-1og10 of the q-value) with each respective environmental variable is show on the y-axis. Chromosomes and cumulative position of SNPs is show on the y-axis. A significance cut-off of 0.05 (red dashed line) was chosen. SNPs depicted in red were also identified by the RDA analysis associated with that environmental variable and present in the outliers detected by pcadapt.
