# Supplementary material for: Adaptive Potential of Syzygium maire, a Critically Threatened Habitat Specialist Tree Species in Aotearoa New Zealand
Source: Evol Appl. 2025 Oct 2;18(10):e70161. doi: 10.1111/eva.70161 (PMC12489745; doi:10.1111/eva.70161)
Supplement: Supplementary file 16 — Table S1: Breakdown of samples by regions, subregions, genetic cluster and sample size for the 269 trees characterized in this study. [file EVA-18-e70161-s013.docx]

**Table S1:** **Breakdown of samples by regions, subregions, genetic cluster and sample size for the 269 trees characterised in this study.**

| **Region** | **Iwi^1^** | **Cluster (MAF0.05)** | **Sample size^2^** | **Subregion** | **Number of sampling sites** | **Sample size^2^** |
| --- | --- | --- | --- | --- | --- | --- |
| Northland | Te Roroa | 3 | 41 (39) | Waipoua | 6 | 41 (39) |
| Bay of Plenty | Ngā Pōtiki, Ngāi Te Rangi, Ngāti Pūkenga, Ngāti Ranginui, Waitaha | 2 | 16 (8) | Tauranga | 4 | 16 (8) |
| Taranaki | Ngati Tama | 4 | 48 (46) | New Plymouth | 1 | 15 |
|  | Taranaki iwi, Ngā Ruahine |  |  | Stratford | 2 | 33 (31) |
| Manawatū | Rangitāne o Manawatū, Rangitāne o Tāmaki nui-ā-Rua | 5 | 29 (20) | Manawatū | 3 | 29 (20) |
| Greater Wellington | Āti Awa ki Whakarongotai, Ngāti Kahungunu ki Wairarapa, Rangitāne o Wairarapa, Ngāti Toa Rangatira, Taranaki Whānui ki Te Upoko o Te Ika, Te Āti Awa, Muaūpoko | 5 | 111 (79) | Kāpiti Coast | 2 | 22 |
|  |  |  |  | Wairarapa | 4 | 41 (30) |
|  |  |  |  | Hutt Valley | 4 | 35 (20) |
|  |  |  |  | Wellington | 1 | 13 (7) |
| Marlborough | Ngāti Toa Rangatira, Te Ātiawa o Te Waka-a-Māui, Rangitāne o Wairau, Ngāti Kuia | 1 | 24 (11) | Marlborough (East) | 3 | 4 (3) |
|  |  |  |  | Marlborough (West) | 2 | 20 (8) |
| Total |  |  | 269 (203) |  | 32 | 269 (203) |

^1^based on the boundaries determined for which iwi exercise kaitiakitanga for the purposes of the Resource Management Act 1991

^2^Numbers in parentheses indicate sample sizes upon removal of related individuals
