# Supplementary material for: Adaptive Potential of Syzygium maire, a Critically Threatened Habitat Specialist Tree Species in Aotearoa New Zealand
Source: Evol Appl. 2025 Oct 2;18(10):e70161. doi: 10.1111/eva.70161 (PMC12489745; doi:10.1111/eva.70161)
Supplement: Supplementary file 17 — Table S2: Variant calling statistics for single nucleotide polymorphisms (SNPs) according to: varying minor allele frequency (MAF), linkage disequilibrium (LD) and outlier filters. [file EVA-18-e70161-s015.docx]

**Table S2: Variant calling statistics for single nucleotide polymorphisms (SNPs) according to: varying minor allele frequency (MAF), linkage disequilibrium (LD) and outlier filters.**

|  | **SNPs** | **SNPs/kilobase** | **Mean depth** | **% missing data** | **Analyses** |
| --- | --- | --- | --- | --- | --- |
| No filters | 3,770,953 | 9.43 | 6.26 |  |  |
| MAF0.00 - LD | 957,330 | 2.39 | 6.77 | 1.08 | Kinship, summary statistics |
| MAF0.05 - LD | 188,131 | 0.47 | 6.75 | 1.16 | DAPC, admixture, summary statistics, GEA, K-means |
| MAF0.05 - LD - outliers | 126,386 | 0.32 | 6.51 | 1.38 | Admixture, summary statistics, GEA |
