# Supplementary material for: Adaptive Potential of Syzygium maire, a Critically Threatened Habitat Specialist Tree Species in Aotearoa New Zealand
Source: Evol Appl. 2025 Oct 2;18(10):e70161. doi: 10.1111/eva.70161 (PMC12489745; doi:10.1111/eva.70161)
Supplement: Supplementary file 18 — Table S3: Pairwise F ST and confidence intervals for two datasets, one with and one without outlier alleles. [file EVA-18-e70161-s003.docx]

**Table S3: Pairwise F_ST_ and confidence intervals for two datasets, one with and one without outlier alleles**

| **Population comparison** | **+ outliers** | | | - **outliers** | | |
| --- | --- | --- | --- | --- | --- | --- |
|  | **F_ST_** | **F_ST_ 2.5% CI** | **F_ST_ 97.5% CI** | **F_ST_** | **F_ST_ 2.5% CI** | **F_ST_ 97.5% CI** |
| NOR-BOP | 0.13 | 0.13 | 0.13 | 0.03 | 0.03 | 0.03 |
| NOR-TAR | 0.11 | 0.11 | 0.11 | 0.03 | 0.02 | 0.03 |
| NOR-MAN | 0.15 | 0.15 | 0.15 | 0.04 | 0.04 | 0.04 |
| NOR-GWE | 0.14 | 0.13 | 0.14 | 0.03 | 0.03 | 0.03 |
| NOR-MAR | 0.29 | 0.29 | 0.29 | 0.07 | 0.07 | 0.07 |
| BOP-TAR | 0.1 | 0.1 | 0.1 | 0.03 | 0.03 | 0.03 |
| BOP-MAN | 0.14 | 0.14 | 0.15 | 0.04 | 0.04 | 0.04 |
| BOP-GWE | 0.13 | 0.12 | 0.13 | 0.03 | 0.03 | 0.03 |
| BOP-MAR | 0.32 | 0.32 | 0.33 | 0.06 | 0.06 | 0.06 |
| TAR-MAN | 0.11 | 0.1 | 0.11 | 0.03 | 0.02 | 0.03 |
| TAR-GWE | 0.1 | 0.1 | 0.1 | 0.02 | 0.01 | 0.02 |
| TAR-MAR | 0.24 | 0.23 | 0.24 | 0.05 | 0.05 | 0.05 |
| MAN-GWE | 0.05 | 0.04 | 0.05 | 0.01 | 0.01 | 0.01 |
| MAN-MAR | 0.22 | 0.22 | 0.22 | 0.04 | 0.04 | 0.04 |
| GWE-MAR | 0.19 | 0.18 | 0.19 | 0.04 | 0.04 | 0.04 |
