# Supplementary material for: Adaptive Potential of Syzygium maire, a Critically Threatened Habitat Specialist Tree Species in Aotearoa New Zealand
Source: Evol Appl. 2025 Oct 2;18(10):e70161. doi: 10.1111/eva.70161 (PMC12489745; doi:10.1111/eva.70161)
Supplement: Supplementary file 19 — Table S4: Partial redundancy analysis (pRDA) accounting for the effect of climate and neutral genetic structure. [file EVA-18-e70161-s014.docx]

**Table S4: Partial redundancy analysis (pRDA) accounting for the effect of climate and neutral genetic structure.**

| **Model** | **Inertia** | **R^2^** | **p (>F)** | **Proportion of explainable variance** | **Proportion of total variance** |
| --- | --- | --- | --- | --- | --- |
| Full model: F ~ clim. + struct. | 391 | 0.132 | 0.001 | 1 | 0.13 |
| Pure climate: F ~ clim. \| (struct.) | 163 | 0.055 | 0.001 | 0.42 | 0.05 |
| Pure structure: F ~ struct. \| (clim.) | 139 | 0.047 | 0.001 | 0.36 | 0.05 |
| Confounded climate/structure | 89 |  |  | 0.23 | 0.03 |
| Total unexplained | 2,576 |  |  |  | 0.87 |
| Total inertia | 2,967 |  |  |  | 1 |

Inertia = variance
