# Supplementary material for: Adaptive Potential of Syzygium maire, a Critically Threatened Habitat Specialist Tree Species in Aotearoa New Zealand
Source: Evol Appl. 2025 Oct 2;18(10):e70161. doi: 10.1111/eva.70161 (PMC12489745; doi:10.1111/eva.70161)
Supplement: Supplementary file 20 — Table S5: Number of SNPs associated with various environmental variables for less (q value < 0.05 and SD 2.5) and more (q‐value < 0.1 and SD 3) stringent threshold filters for LFMM and RDA analysis respectively, and overlap with SNPs identified as PCA outliers. [file EVA-18-e70161-s009.docx]

**Table S5: Number of SNPs associated with various environmental variables for less (q value < 0.05 and SD2.5) and more (q-value < 0.1 and SD3 ) stringent threshold filters for LFMM and RDA analysis respectively, and overlap with SNPs identified as PCA outliers.**

| **Covariate** | **LFMM** | **RDA** | **Shared** | **Common with Pcadapt** |
| --- | --- | --- | --- | --- |
| **FDR0.1, SD2.5** |  |  |  |  |
| solRad_meanAnn | 461 | 827 | 39 | 27 |
| temp_meanAnn | 228 | 1,378 | 53 | 26 |
| precip_seasonality | 1,241 | 901 | 125 | 95 |
| temp_annRange | 477 | 1,264 | 133 | 83 |
| waterBalance_annDeficit | 392 | 1,744 | 182 | 91 |
| humidity_meanAnn | 55 | 965 | 19 | 18 |
| soil_acidP | 101 | 616 | 25 | 7 |
| soil_calcium | 182 | 2,752 | 95 | 61 |
| soil_drainage | 2 | 377 | 0 | 0 |
| topo_position | 293 | 1,547 | 17 | 9 |
| Total | 3,432 | 12,371 | 688 | 417 |
|  |  |  |  |  |
| **FDR0.05, SD3** |  |  |  |  |
| solRad_meanAnn | 298 | 270 | 15 | 9 |
| temp_meanAnn | 118 | 509 | 30 | 13 |
| precip_seasonality | 791 | 375 | 48 | 40 |
| temp_annRange | 262 | 530 | 53 | 33 |
| waterBalance_annDeficit | 163 | 703 | 66 | 38 |
| humidity_meanAnn | 20 | 345 | 3 | 3 |
| soil_acidP | 69 | 253 | 8 | 0 |
| soil_calcium | 45 | 1,182 | 19 | 12 |
| soil_drainage | 2 | 120 | 0 | 0 |
| topo_position | 186 | 655 | 4 | 3 |
| Total | 1,954 | 4,942 | 246 | 151 |
